# Adhesive hydrogels in osteoarthritis: from design to application

**DOI:** 10.1186/s40779-022-00439-3

**Published:** 2023-01-30

**Authors:** Wang-Lin Duan, Li-Ning Zhang, Raghvendra Bohara, Sergio Martin-Saldaña, Fei Yang, Yi-Yang Zhao, Yong Xie, Ya-Zhong Bu, Abhay Pandit

**Affiliations:** 1 Institute of Medical Engineering, Department of Biophysics, School of Basic Medical Sciences, Health Science Center, Xi’an Jiaotong University, Xi’an, 710061 China; 2 Department of Rehabilitation Medicine, the First Medical Center, Chinese PLA General Hospital, No.28 Fuxing Road, Haidian District, Beijing, 100853 China; 3 CÚRAM, SFI Research Centre for Medical Devices, University of Galway, Galway, H91 TK33 Ireland; 4 Beijing National Laboratory for Molecular Sciences, State Key Laboratory of Polymer Physics and Chemistry, Institute of Chemistry, Chinese Academy of Sciences, Beijing, 100190 China; 5 School of Chemical Sciences, University of Chinese Academy of Sciences, Beijing, 100049 China; 6 Department of Orthopedics, the Fourth Medical Center, Chinese PLA General Hospital, Beijing, 100853 China; 7 National Clinical Research Center for Orthopedics, Sports Medicine and Rehabilitation, Beijing, 100853 China

**Keywords:** Adhesive hydrogel, Osteoarthritis (OA), Functional additives, Cartilage regeneration, Interdisciplinary therapy

## Abstract

Osteoarthritis (OA) is the most common type of degenerative joint disease which affects 7% of the global population and more than 500 million people worldwide. One research frontier is the development of hydrogels for OA treatment, which operate either as functional scaffolds of tissue engineering or as delivery vehicles of functional additives. Both approaches address the big challenge: establishing stable integration of such delivery systems or implants. Adhesive hydrogels provide possible solutions to this challenge. However, few studies have described the current advances in using adhesive hydrogel for OA treatment. This review summarizes the commonly used hydrogels with their adhesion mechanisms and components. Additionally, recognizing that OA is a complex disease involving different biological mechanisms, the bioactive therapeutic strategies are also presented. By presenting the adhesive hydrogels in an interdisciplinary way, including both the fields of chemistry and biology, this review will attempt to provide a comprehensive insight for designing novel bioadhesive systems for OA therapy.

## Background

Osteoarthritis (OA) is the most common type of degenerative joint disease affecting 7% of the global population and more than 500 million people worldwide [1–3]. The number of people affected by OA continues to increase because of rising life expectancy [4]. Over the last three decades, the number of OA-affected people have risen by 48%. This makes it the 15th most significant cause of disability worldwide, imposing a high cost on patients and the healthcare system. The statistics are particularly alarming for women, some racial and ethnic groups, and for individuals of lower socioeconomic status [5]. In addition to suggestions on moderate exercise and a healthy diet, current clinical therapeutic procedures vary from oral drug administration and intra-articular injection to surgery. Intra-articular injection mainly involves the local administration of functional components, like dexamethasone (DEX), growth factors or lubricants [6–8]. However, oral drug taking causes systemic toxicity to the gastrointestinal and cardiovascular systems [3]. The intra-articular injection of functional components also has limited efficacy because of their fast clearance and the ease of moving to other places due to joint motion [9, 10]. When the OA induces cartilage defects, patients are more likely to undergo surgeries, including microfracture, cell transplantation, and tissue transplantation [11, 12]. These surgical procedures, however, are annexed with problems such as a limited number of donors, low chondrogenic efficiency, and poor integration with surrounding cartilage tissue [13].

Hydrogels are crosslinked polymer networks with a high-water content, and can be fabricated to simulate the physicochemical properties of cartilage tissue [14]. These approaches provide a new avenue for research in cartilage regeneration [15]. There are two frontline trends of hydrogel-based therapies for OA therapy [15, 16]. The first one is using a hydrogel-based tissue scaffold implants for cartilage tissue regeneration, which falls within the scope of tissue engineering [17–19]. The second one is to develop hydrogel-based systems for delivering functional additives to the injured cartilage and maintain the stability of those active ingredients at the site over a prolonged time. Both strategies aim at promoting tissue regeneration through different mechanisms. However, they have significant challenges: achieving a stable integration of those delivery systems or appropriate implantation into the injury site [12]. The newly formed cartilage surrounding the scaffold will lack stability without adequate bonding, thus unable to integrate with the host tissue. This would result in the failure of functions in synovial joints under cyclic compressive and shear stress [20, 21]. Furthermore, it also leads to fibrosis between the host implant and cartilage, causing failure of the neo-cartilage to integrate with native cartilage [22]. Moreover, the interfaces between the hydrogel systems and wounds easily disjoin. Resultantly, functional additives do not remain stable on the site, thereby decreasing their efficacy.

Adhesive hydrogels with inherent tissue adhesiveness have immense potential for OA therapy. They can stay stable at the site where they are applied [23]. Various methods of fabricating adhesive hydrogels have already been used for wound closure, tissue sealing, and medical device fixation [23, 24]. Thus, applying adhesive hydrogel in OA therapy is an excellent way to overcome the weak integration between biomaterials and cartilage tissue. There is an increasing interest in using adhesive hydrogels as tissue scaffolds or as delivery systems in OA therapy [20, 25–27]. Few reports have summarized the commonly used adhesive hydrogels strategies for OA treatment, although it is widely understood that proper integration between implants and cartilage tissue in OA therapy is critical [20, 26, 28].

This review provides an overview of OA treatment using adhesive hydrogels. This includes the commonly used adhesion mechanisms and compounds in adhesive hydrogels. It is generally recognized that the ability to modulate biological functions is essential for tissue regeneration. Attempts to understand the biological functions of adhesive hydrogels are also summarized. These features result from the functional components of the adhesive hydrogels or are exhibited by the functional additives. Finally, the future trends of adhesive hydrogels in OA therapy are presented from both material and clinical perspectives. This review can facilitate innovations in OA therapy by clarifying adhesive hydrogels’ chemical and biological functions.

## OA pathophysiological features

**Fig. 1:**
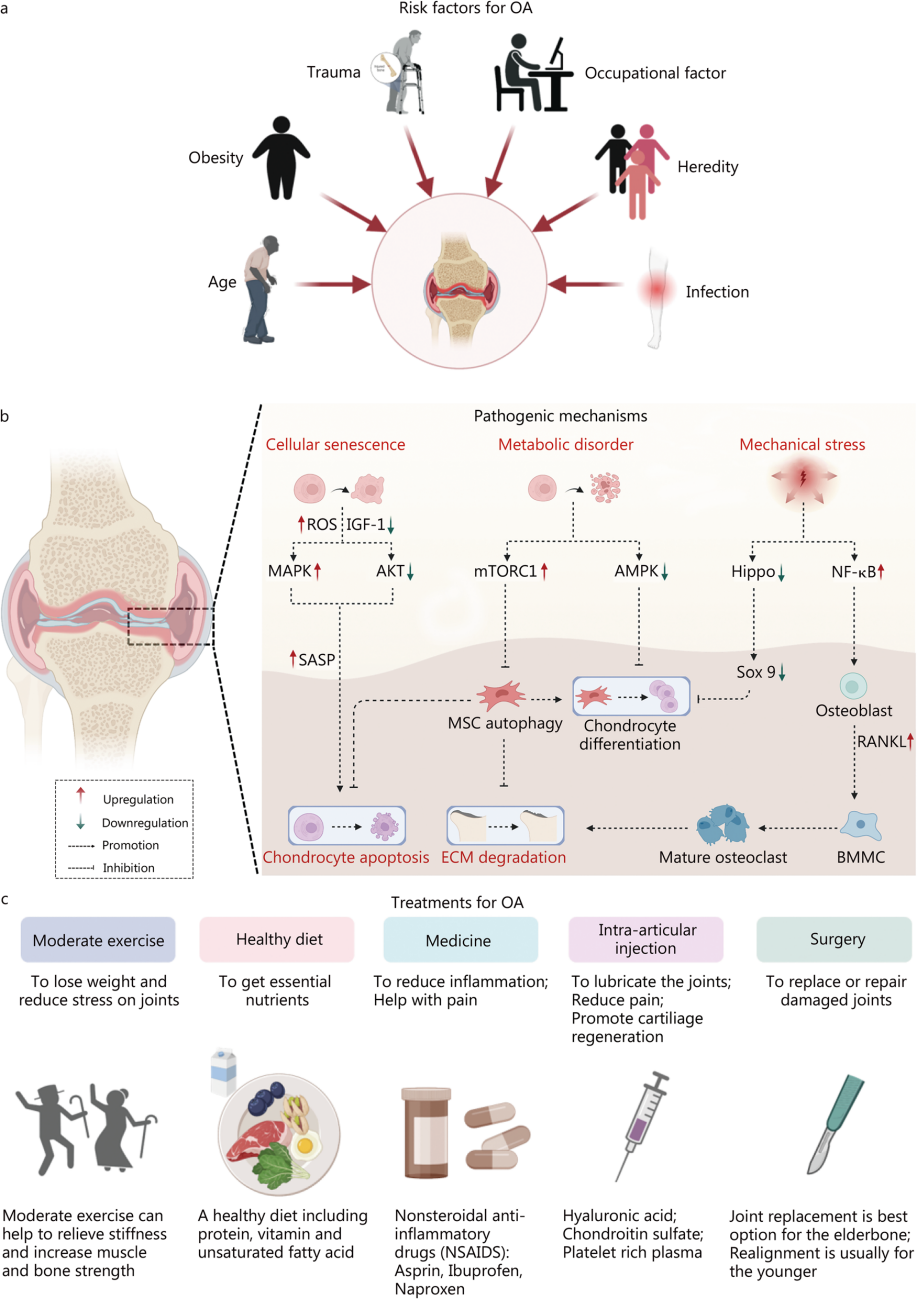
Risk factors, pathogenic mechanisms, and common treatments for OA. **a** Common risk factors that can lead to OA include aging, obesity, trauma, overuse due to occupational reasons, heredity, and infection. **b** Common pathophysiological changes in OA progress. Pathogenic pathways include MAPK, AKT, mTORC1, AMPK, Hippo, NF-κB, etc., regulated by cellular senescence, metabolic disorder and mechanical stress. They have shown to accelerate OA progress thorough chondrocyte apoptosis and ECM degradation. **c** The indications for OA therapy include moderate exercise, a healthy diet, medicine, intra-articular injection of functional components, and surgery. OA osteoarthritis, IGF-1 insulin-like growth factor-1, MAPK mitogen-activated protein kinase, AKT serine/threonine kinase Akt, also known as protein kinase B, SASP senescence-associated secretory phenotype, mTORC1 mammalian target of rapamycin complex 1, MSC mesenchymal stem cell, ECM extra cellular matrix, AMPK adenosine 5’-monophosphate (AMP)-activated protein kinase, Sox 9 SRY-related high mobility group-box 9, NF-κB nuclear factor kappa-B, RANKL receptor activator of NF-κB ligand, BMMC bone marrow mononuclear cell. It was created utilizing the templates on BioRender.com as a reference

OA is an abnormal molecular disorder of joint tissue followed by physiologic derangements, mainly resulting from age, obesity, trauma, occupational joint overuse, heredity and infections (Fig. 1a) [29]. The pathology of OA is characterized by cartilage degradation, subchondral bone remodeling and joint inflammation, which culminates in the narrowing of the joint space, formation of osteophytes, chronic pain and loss of normal joint function [30, 31]. Figure 1b summarizes three common pathogenic mechanisms of OA and the related signaling pathways, including cellular senescence, metabolic disorder and mechanical stress [32, 33].

Chondrocyte senescence plays a considerable role in the impaired integrity and function of the cartilage. The accumulation of senescent cells within joint tissue results in the dysfunction of articular cartilage homeostasis [34]. It is also involved in the overproduction of reactive oxygen species (ROS) [35], and thereby induces cumulative DNA damage and oxidative stress via the activation of the p38 mitogen-activated protein kinase (MAPK) signaling [36, 37]. In addition, the downregulation of survival promoting insulin-like growth factor-1 (IGF-1)-mediated RACα serine/threonine protein kinase (AKT) amplifies the expression of primary pro-inflammatory mediators of OA [38], including prostaglandin E2 (PGE2), and inducible nitric oxide synthase (iNOS), collectively known as the senescence-associated secretory phenotype (SASP). These factors contribute to chondrocyte apoptosis and drive further positive feedback of senescence [39].

In addition to the aging phenotype, OA pathogenesis is also caused by metabolic disorders. The energy metabolism in joint tissue switches from oxidative phosphorylation to anaerobic glycolysis when exposed to nutrient stress [40]. The activity of the mechanistic target of rapamycin complex 1 (mTORC1) is upregulated which reduces mesenchymal stem cell (MSC) autophagy and its anti-catabolic effect on chondrocyte and extracellular matrix (ECM). Besides, 5'-monophosphate (AMP)-activated protein kinase (AMPK) signaling activity is downregulated, disrupting the differentiation process of chondrocytes from MSC [41, 42].

Cartilage biomechanical function depends on ECM, which responds to normal weight-bearing forces [43]. Aberrant mechanical stress from obesity or joint injury is expected to contribute to the upregulation of the nuclear factor kappa-B (NF-κB) pathway [44], thereby regulating the ECM degradation via osteoclastic resorption. This is promoted by upregulation of osteoblastic receptor activator of NF-κB ligand (RANKL) that mediates bone marrow mononuclear cell (BMMC) differentiation. The loss of cartilage integrity leads to unstable mechanical condition and further excessive loading. Then, the deactivated Hippo loses control over YAP/TAZ, which translocates into the nucleus to develop transcript factor Sox 9 for chondrocyte differentiation [43]. All these mediators disrupt the osteoimmune environment in joint tissue [32] which results in the progressive destruction of articular cartilage and sclerotic bone formation [45].

The clinical indications for OA therapy include moderate exercise, healthy diet, medicines, intra-articular injection, and surgery (Fig. 1c). The oral uptake of medicine and intra-articular injection are the most often used methods. However, the systematic toxicity caused by available drugs due to high dose needed and their fast clearance limits their effects. Therefore, the adhesive hydrogels will be relevant for future therapeutic strategies, which retain the functional components and ensure a stable integration. Subsequently, a stable integration will also improve the stability of mechanical restoration. In addition, considering the complex pathogenic features of OA (Fig. 1b), adhesive hydrogels must have biological functions to achieve desirable therapeutic effects.

## Adhesion mechanisms of hydrogel in OA

**Fig. 2:**
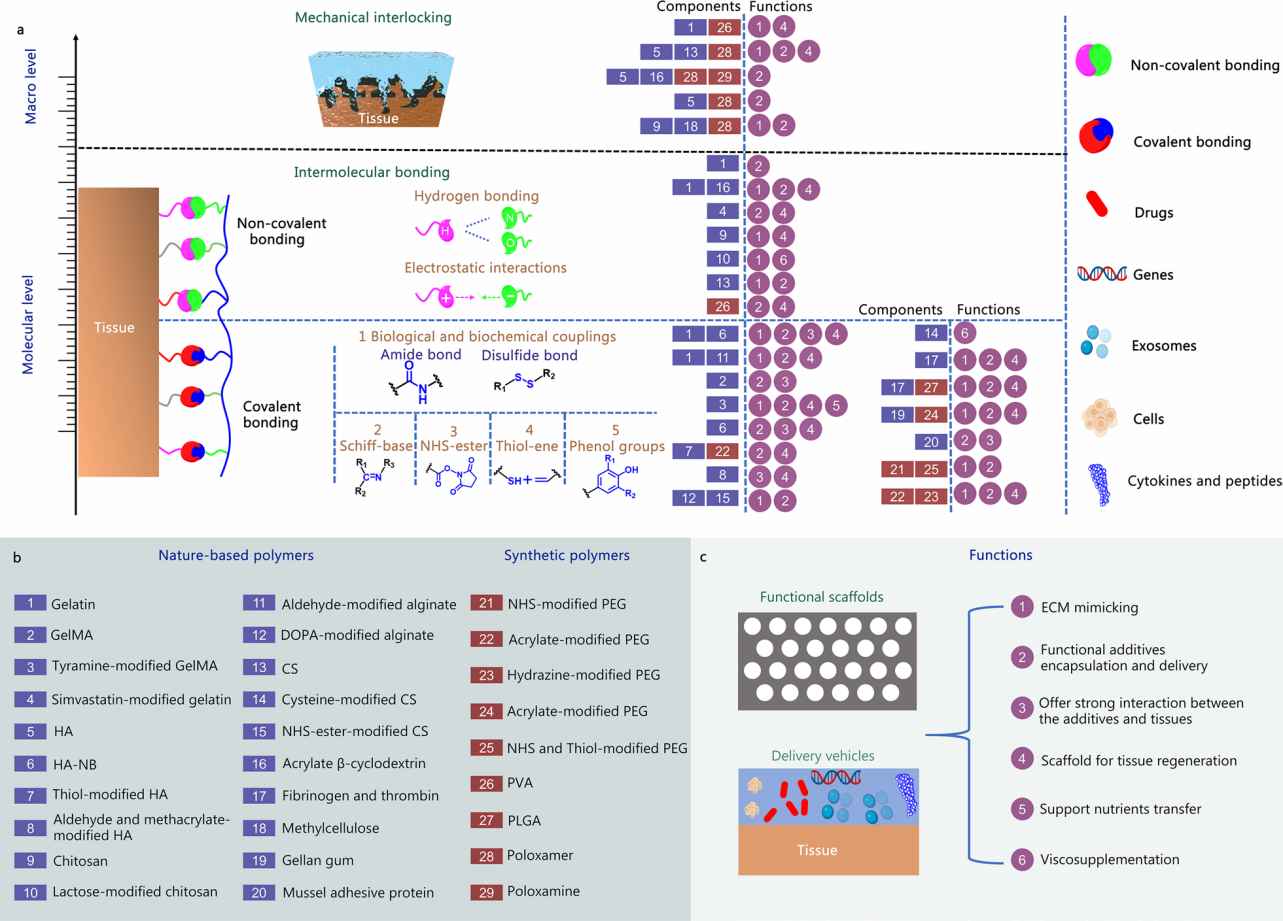
Main adhesion mechanisms, components, and potential functions of adhesive hydrogels in OA therapy. **a** Two common adhesion mechanisms behind adhesive hydrogels for OA therapy. The first one is mechanical interlocking formed on macro level. Irregular tissue or pores are needed for relatively weak adhesiveness. The second is intermolecular bonding including non-covalent and covalent ones formed on molecular level. Non-covalent bonding includes hydrogen bonding from a hydrogen atom covalently bonded to electronegative atom such as N and O, and electrostatic attraction between two oppositely charged molecules. Covalent bonding includes biological and biochemical couplings (Amide bond and Disulfide bond), Schiff-base, NHS-ester, Thiol-ene and Phenol groups. **b** A list of components for fabricating adhesive hydrogels, including nature-based polymers (Gelatin, HA, CS and their modification products, etc.) and synthetic polymers (PVA, PLGA, various modification products of PEG, etc.). **c** Potential functions of adhesive hydrogels in OA therapy. OA osteoarthritis, CS chondroitin sulfate, GelMA methacrylated gelatin, HA hyaluronic acid, HA-NB o-nitrobenzyl alcohol-modified HA, PEG polyethylene glycol, PVA polyvinyl alcohol, PLGA polylactic-*co*-glycolic, ECM extra cellular matrix. It was created utilizing the templates on BioRender.com as a reference

Adhesive hydrogels, because of their inherent adhesion toward the tissue, can retain stability where applied and extend the functional duration of loaded additives in OA treatment. The review’s first aim is to understand the adhesion mechanisms behind different adhesive hydrogels for OA. Because of the cartilage’s structure, materials’ adhesion to tissue’s surfaces can be categorized depending on the scale, which includes mechanical interlocking on macro level and intermolecular bonding on molecular level (Fig. 2a) [46].

### Mechanical interlocking

As shown in Fig. 2a, the term mechanical interlocking refers to how adhesive hydrogels infiltrate pores and irregularities on tissue surfaces. It relates to the microscopic roughness of the tissue surface [47]. Getting suitable tissue adhesion through mechanical interlocking in healthy cartilage tissue is challenging as tissue structure is flat, slippery, and firm. However, this structure becomes rougher and uneven due to pathogenic changes, thus providing pores and irregularities for mechanical interlocking. Thermo-responsive hydrogels commonly use this mechanism. Before application, these hydrogels are in a liquid state. Once applied, they flow through the irregularities of the tissue surface and undergo phase transition due to changes in temperature under physiological conditions. Li et al. [48] fabricated a Pluronic F127-based thermo-sensitive hydrogel for OA-induced cartilage injury. The hydrogel provides strong adhesion for long-term retention because of the temperature-induced in situ gelation. Rey-Rico et al. [49] used Pluronic F68/Tetronic 908 with hyaluronic acid (HA)/chondroitin sulfate (CS) to generate a thermo-sensitive hydrogel for recombinant adeno-associated virus (rAAV) vector delivery in cartilage regeneration.

*Pros and cons*: Mechanical interlocking is a straightforward way to achieve tissue adhesion. This mechanism requires the fluid to flow into the irregular structure before gelation occurs. The injectable hydrogels can be in the liquid state upon injection and become solid for fixation. Most in situ injectable hydrogels can achieve adhesion through mechanical interlocking by adjusting the gelation behaviour [46, 47]. However, the mechanical interlocking relies fundamentally on the topological structure of the cartilage tissue. It is thus influenced by the tissue’s condition and stage of disease and varies from tissue to tissue. Furthermore, the adhesive hydrogels and tissue do not integrate at molecular level due to the lack of intramolecular interactions. Therefore, mechanical interlocking hinders in achieving the high adhesion strength around cartilage tissue.

### Intermolecular bonding

Compared with mechanical interlocking on macro scale, intermolecular bonding relates to molecular interactions resulting from forces and bonds of atoms/molecules between adhesive hydrogels and the tissue surfaces. These bonds can be primary or secondary forces, including non-covalent and covalent ones. Intermolecular bonding is the primary adhesion mechanism for adhesive hydrogels in OA therapy.

#### Non-covalent bonding

The non-covalent bond is a bond between macromolecules which does not share pairs of electrons [50]. It is paramount in maintaining the structure of tissue proteins which have many sites for developing non-covalent bonding. Non-covalent bonding includes hydrogen bonding, electrostatic interactions and van der Waals interactions. Among them, hydrogen bonding and electrostatic interactions are the most used adhesion mechanism in cartilage tissue adhesive hydrogels, so we only discuss these two mechanisms below.

*Hydrogen bonding*: The hydrogen bonding is a particular type of dipole-dipole attraction between molecules. It results from the attractive force between a hydrogen atom covalently bonded to a strongly electronegative atom such as N, O, or F or another highly electronegative atom [51]. In biological tissue, hydrogen bonding often occurs between N and O [52]. Typically, the hydrogen bonding in adhesive hydrogels for OA therapy comes from polymers with plenty of hydroxyl groups, such as polyvinyl alcohol (PVA) [53–55].

*Electrostatic interactions*: Electrostatic interactions include attractive and repulsive interactions. The engaging interactions between two oppositely charged molecules lead to adhesion. Polysaccharides in OA therapy, including chitosan and CS, interact with cartilage tissue through electrostatic interactions [56]. Gelatin is also a polymer that can bind with tissue through electrostatic interactions. JointRep® (Oligo Medic Inc., Laval, Quebec, Canada) is a bioadhesive gel designed for cartilage regeneration. It possesses good tissue adhesion to the cartilage because of the ionic bonds formed between chitosan and the tissue [57].

*Pros and cons*: The non-covalent bonding is advantageous because of less chemical modifications or chemical crosslinkers. Biological properties of rare materials are less likely to change and toxic components are avoided [58]. Moreover, although rarely mentioned in OA therapy, the reversibility of non-covalent crosslinking enables repeated attachment to the biological tissue [59]. However, the overall adhesion strength is weak because of low bond energy of non-covalent crosslinking [60].

#### Covalent bonding

Covalent interactions produce the strongest bonding in nature [61]. As a result, adhesion through covalent bonds is often very stable. The commonly used covalent strategies in OA therapy are listed below.

*Biological and biochemical couplings*: Biological and biochemical couplings are the molecule-molecule interactions involved in the daily metabolic activity of organisms [62]. There are two typical examples. The first one is the last step of clotting cascades involved in the fibrinogen-thrombin interactions of Fibrin glue. Fibrin glue mainly contains two components, fibrinogen and thrombin [63]. Upon mixing, thrombin catalyzes the formation of amide bonds between glutamine and lysine amino acids in fibrin polymer chains. The fibrin polymer also crosslinks with the surrounding tissue causing adhesion [64]. Li et al. [65] used polylactide-*co*-glycolide (PLGA)/Fibrin glue to develop a system for combining gene therapy with tissue engineering. The system delivered both genes and MSCs to cartilage injuries. Fibrin gel not only offered tissue adhesion but also enabled slower gene release, which led to a higher cell loading density. It was found that transforming growth factor-β1 (TGF-β1) expression was upregulated by Fibrin gel. After getting treated for 12 weeks using this system, full-thickness defects of cartilage were resurfaced by neo-tissue with a structure like that of surrounding tissue.

Another commonly existing crosslinking in biology is the disulfide bond formed between two thiol groups, which maintains the stereo structures of native proteins. Because of the thiol groups in native protein, thiol-modified polymers show tissue adhesion via disulfide bond formation. This is extremely important for mucoadhesion. Suchaoin et al. [66] used cysteine (cys) to modify CS for fabricating the bioadhesive agent for OA therapy. The CS-cys adhesive showed a 5.37-fold increase in adhesion strength when tested with porcine articular cartilage. The adhesive demonstrated cytocompatibility against Caco-2 cells and rat primary articular chondrocytes. CS-cys might be a promising intra-articular agent for OA treatment because of the increased bioadhesive properties.

*Schiff-base*: Schiff-base is formed by the reaction between aldehyde/ketone groups and amino groups [67, 68]. As many amino groups are there in tissue proteins, modifying compounds with aldehyde groups is preferred to fabricate hydrogels that can adhere to cartilage tissue. Chen et al. [22] made aldehyde and methacrylate-modified HA. The increased tissue anchoring with the Schiff-base promoted integration between neo-cartilage and host tissue, significantly improving the cartilage regeneration. O-nitrobenzyl alcohol-modified HA (HA-NB) conjugate generates aldehyde groups upon light irradiation through a photo-triggered imine-crosslinking reaction where o-nitrobenzene is converted to o-nitrosobenzaldehyde upon 365 nm illumination. Liu et al. [69] used HA-NB with platelet-rich plasma (PRP) to generate PRP-loaded o-nitrobenzyl alcohol-modified HA adhesive hydrogel to overcome the unstable fixation and burst release of PRP. In vivo studies proved that PRP-loaded o-nitrobenzyl alcohol-modified HA adhesive hydrogels achieved higher therapeutic efficacy than thrombin-activated PRP hydrogels.

*N-hydroxysuccinimide-ester (NHS-ester)*: NHS-ester is an active ester which is highly reactive toward nucleophilic attack. Together with N-hydroxysulfosuccinimide-ester (Sulfo-NHS-ester), they show high reactivity for primary amines and thiol groups under mild conditions. They are widely used in several bioconjugation techniques, like peptide synthesis, fluorescence labelling, etc. [70, 71]. They have also been used in fabricating adhesive hydrogels for cartilage regeneration because of their strong reaction toward the primary amines and thiol groups. The commonly used NHS-ester-containing polymer is polyethylene glycol (PEG). With 4-arm-PEG-NHS and gelatin microgels, Li et al. [25] fabricated NHS-treated assembled (NHSA) microgels, a 3D construct with tissue adhesiveness to the cartilage tissue. NHSA-microgels upregulated chondrogenic markers at the gene and glycosaminoglycan (GAG) expression levels. Moreover, hyaline-like cartilage tissue was formed in NHSA-microgels.

*Thiol-ene*: Thiol-ene is a type of click chemistry having high thermodynamic driving forces and rapid reaction. As there are thiol groups in the tissue proteins, ene-modified polymers can also react with them to provide tissue adhesion [72]. Although MSCs are promising in repairing cartilage injury, the methods for delivering and maintaining them on-site remain to be devised. Li et al. [73] used acrylate-modified PEG and thiol-modified HA to fabricate hydrogel for MSCs delivery. The hyper-branched structure of the acrylate-modified PEG functioned as the adhesive precursor because of forming links with tissue via thiol-ene reaction. MSCs were loaded in the adhesive hydrogel for better therapeutic outcomes. It could significantly repair full-thickness cartilage defects in rat model after 8 weeks of implantation [73]. Using dithiobis (propanoic dihydrazide), thiol groups can be introduced into the polysaccharide. Li et al. [72] synthesized an adhesive hydrogel by thiol-modified CS and acrylate-containing hyperbranched PEG. MSCs loading in the hydrogel had increased cell viability and improved chondrogenesis. Additionally, the adhesive hydrogels showed anti-inflammatory response because of the CS, thus suggesting great promise in cartilage tissue engineering.

*Phenol groups*: Phenol groups are popular candidates for wet adhesion including monophenol-based tyrosine, maritime species-inspired dopamine (DOPA) chemistry, and plant-inspired polyphenols such as tannic acid and pyrogallol [23]. Although cartilage tissue engineering products have been authorized for clinical usage, weak tissue adhesion is still a problem. Feng et al. [26] fabricated a dynamic nanocomposite hydrogel with microporosity, injectability and tissue adhesive properties that target OA-induced cartilage injury. DOPA-modified HA was used to coat the hydrogel systems via dynamic crosslinking to promote tissue adhesion. Kartogenin and bone mesenchymal stem cells (BMSCs) were loaded into the adhesive hydrogels for a better therapeutic outcome. Animal studies revealed that the functional additives-loaded adhesive systems promoted the cartilage regeneration in which the newborn cartilage presented typical characteristics of articular cartilage. Other adhesion groups have also been used with phenol groups to further increase the adhesion strength. Zhang et al. [74] fabricated the mussel-inspired adhesive and injectable hydrogels for cartilage regeneration. DOPA-modified alginate was used to introduce wet adhesion in their design. The adhesion strength was further enhanced by using NHS-ester-modified CS and regenerated silk fibroin containing lysine and tyrosine. The resulting adhesive hydrogel provided comparative lap shear strength of 120 kPa. Later, the exosomes were loaded into the adhesive hydrogel, suggesting that BMSCs were recruited to the adhesive hydrogel and neo-cartilage.

*Pros and cons*: Covalent bonding is the most robust bonding for the interactions in tissue adhesion. Among them, biological interactions, Schiff-base and NHS-ester are the strategies employed in currently used clinical products, like Tisseel® (Biological interactions), Coseal® and Duraseal® (NHS-ester), Bioglue® (Schiff-base). However, although they generate strong interactions with host tissue, none of the reports show a breakthrough in achieving very large adhesive strength. Furthermore, some require complex preparatory steps and even special storage conditions, compromising their cost-effectiveness. NHS-ester is easy to hydrolyze, so it must be stored in powder form and in dry environment. Phenol groups easily get oxidized and thus require controlled storage environment. Additionally, the colour change after oxidation and the potential neurological effects of phenol-based strategies may hinder their use in tissue adhesion.

## Components of the adhesive hydrogels

Components of adhesive hydrogels play a vital role in the end application. Several materials have been used to fabricate adhesive hydrogels for OA therapy. Based on their properties, these materials are classified into two categories: nature-based polymers and synthetic polymers (Fig. 2b). These materials are discussed in detail below.

### Nature-based polymers

Nature-based polymers extracted from plants, animals, or microorganisms have been used in biomedical applications due to their biocompatibility and biofunctions [75]. Hydrogels prepared from nature-based polymers have similar advantages as natural ECM [76, 77]. They can improve cellular behavior and are thus used in tissue regeneration [78, 79]. Here, the natural components of adhesive hydrogels commonly used in OA treatment are discussed below.

*Hyaluronic acid*: HA, a linear polysaccharide, is one of the major components of cartilage ECM and has been widely studied in cartilage regeneration and OA therapy [80]. It has lubrication function and is involved in various cellular processes, like modulating the inflammatory response, cell adhesion, migration, proliferation, differentiation, angiogenesis, and tissue regeneration [81, 82]. In clinic, HA solutions are given weekly for viscosupplementation and pain relief [83]. However, since 2013, the American Academy of Orthopaedic Surgeons no longer recommends intra-articular injection of HA for OA treatment due to its negligible effects compared to the control groups [84]. Generally, HA solutions consist of non-crosslinked HA that do not maintain the desired volume and structural integrity, leading to limited retention time and reducing their ability to deliver functional additives. As a result, HA is chemically modified to fabricate hydrogels [85]. However, although HA has shown to have mucoadhesive properties, the resulting HA-based hydrogel shows weak adhesion to the tissue [86–88]. Various adhesive functional groups have been grafted to HA for raising retention time and therapeutic efficacy, e.g., catechol groups [89], methacrylate [90], aldehyde [91], tyramine [92], and o-nitrobenzyl alcohol [69]. The stable adhesion of HA-based adhesive hydrogels significantly promotes integration between neo-cartilage and host tissue, increasing the therapeutic efficacy [22]. Chen et al. [22] used aldehyde groups to modify HA, and photo-crosslinking was employed to generate the adhesive hydrogel. The resulting adhesive hydrogel showed significantly higher adhesion strength than Fibrin glue. In vivo experiments demonstrated that adhesive hydrogels significantly promoted the integration between neo-cartilage and host tissue and improved cartilage regeneration compared to non-adhesive control.

*Alginate*: Alginate, extracted from brown algae or bacteria, has applications in biomedical science and engineering due to its biocompatibility and ease of gelation [93]. Alginate hydrogels have weak adhesion to tissue, so chemical modifications are needed to improve that property [94, 95]. Currently, the most used modification method is the generation of aldehyde groups in alginate polymer chains via oxidation. Subsequently, the adhesive hydrogel is formed by the reaction between aldehyde-modified alginate and another amino-containing crosslinker. Since alginate hydrogels are promising candidates for cell and gene delivery [96–98], alginate-based adhesive hydrogels have also been used to deliver cells for OA treatment and the regeneration of cartilage tissue [99]. Kreller et al. [100] designed an oxidized alginate and gelatin-based 3D printing hydrogel (ADA-GEL) for cartilage tissue engineering in OA treatment. ADA-GEL with shape stability and fidelity could be printed in complex hierarchical scaffolds for cell encapsulation and mimick the intrinsic hierarchical structure of natural articular cartilages, which is promising in OA therapy. In addition to nature-based crosslinkers with amino groups, synthetic polymers having amino groups can also be crosslinked with aldehyde-modified alginate. This further expands the functions of alginate-based adhesive hydrogels. Yan et al. [101] prepared injectable adhesive hydrogels with aldehyde-modified alginate and hydrazide-modified poly(l-glutamic acid). By changing the solid contents and the oxidation degree of alginate, the resulting adhesive hydrogels showed adjustable mechanical properties and degradation rates. Compared with chondrocyte injection alone, the chondrocyte-loading adhesive hydrogel resulted in more cartilage-like tissue with improved ability to maintain the desired shape.

*Chitosan*: The partial deacetylation of chitin results in chitosan formation [102]. Due to its abundance, versatility, biodegradability, biocompatibility and antimicrobial properties, it has been used in tissue engineering and regeneration. Moreover, it also exhibits tissue adhesion, antioxidant properties, antibacterial activity and anticancer effects as the only positively charged naturally occurring polysaccharide [103, 104]. Hoemann et al. [105] used the tissue adhesiveness of chitosan to develop a chitosan-based adhesive hydrogel for cell delivery. The adhesive gel system remained stable for one week after injecting in osteochondral defects of rabbits. Apart from its biological functions, chitosan can be chemically modified to various derivatives, such as thiolated chitosan, hydroxyalkyl chitosan, etc., to further expand its applications [106]. Scognamiglio et al. [107] fabricated lactose-modified chitosan hydrogel through boric acid crosslinking (CTL-hydrogel). The chitosan adhesive hydrogel showed better stability compared with the traditionally administered HA solution, thus providing long-term viscosupplement for OA treatment. Additionally, lactose-modified chitosan has antioxidant properties which make CTL-hydrogel a high-capacity ROS scavenging system in OA therapy.

*Chondroitin sulfate*: CS, a sulfated GAG consisting of N-acetylgalactosamine and glucuronic acid, is a major component of ECM of cartilage tissue, which is inherently anti-inflammatory, antioxidative and anti-apoptotic [108–110]. It contributes to the synthesis of hyaluronan, collagen and glucosamine and inhibits ECM degradation [111]. It has also been used as a dietary supplement for OA for decades to relieve pain and regenerate cartilage. Although CS has tissue adhesion because of its hydroxyl, carboxyl and amide groups, its intrinsic adhesion strength is relatively low [112]. Therefore, CS are chemically modified with tissue adhesive groups (such as thiol and aldehyde) to achieve higher tissue adhesiveness [66]. To increase bonding strength between implants and cartilage tissue, Wang et al. [113] used methacrylate and aldehyde groups to create a CS-based adhesive which chemically bridges the implants and tissue proteins through two-fold covalent link. The adhesive application significantly improved the therapeutic outcome compared with untreated empty cartilage defects after 6 months. The work also showed the importance of implant integration in the repair of cartilage tissue.

*Gelatin*: Gelatin is a natural water-soluble polymer derived from collagen hydrolysis. Collagen can protect against the onset of joint damage through induction and migration of T regulatory cells and the production of anti-inflammatory cytokines [113, 114]. There are collagen-based hydrogels for OA-induced cartilage defects [15]. However, collagen hydrogels usually have weak mechanical properties and degrade rapidly [115]. Moreover, the chemical modification of collagen is complex because of poor water solubility and low thermostability. This may also be why collagen-based adhesive hydrogels have seldom been reported in OA treatment [116].

In contrast, gelatin is widely explored in tissue regeneration and engineering because of its well-proven biocompatibility, biodegradability, low immunogenicity, water solubility and ease of modification [99, 117, 118]. Also, gelatin-based materials show suitable tissue adhesion through electrostatic interactions produced by carboxyl and amino groups. Zhang et al. [28] used gelatin to fabricate microcryogels for MSCs delivery, which enhanced the retention of MSCs in the knee joint of mice compared with MSCs injection alone. However, the non-covalent crosslinking of gelatin alone was still relatively weak. Therefore, gelatin has also been used with other materials of adhesive groups, like aldehyde-containing materials, to fabricate adhesive hydrogels for OA therapy [100, 119]. In addition, the amino and carboxyl groups of gelatin can be easily modified. Lim et al. [20] used tyramine and methacryloyl to modify carboxyl and amine groups for increasing the tissue adhesion of gelatin. Later, they used photo-crosslinking method to fabricate an adhesive hydrogel for cartilage repair. The adhesive hydrogel showed 15-fold increment in the adhesive strength than methacrylated gelatin (GelMA) alone because of the chemical bonding of tyramine to native cartilage proteins. A high collagen type-II/I ratio was observed in articular chondroprogenitor cells encapsulated GelMA-Tyr hydrogel, indicating the chondrogenic phenotype.

*Mussel adhesive proteins (MAP)*: Marine mussels can attach tightly to foreign surfaces in turbulent seawater due to secreted MAP via DOPA-mediated interfacial bonding [120, 121]. These adhesive proteins have been used for wound closure and cell adhesion. [122, 123]. Tissue engineering-based on stem cell therapy for cartilage regeneration in OA has been developed and used for over 20 years. However, low viability and high possibility of injected cells’ dispersion to target defect sites remain challenging. A report showed that adipose stem cells (ASCs) fixed by MAP on focal chondral defect survived longer than those immobilized with Fibrin glue [124]. Ko et al. [125] used MAP-based adhesive to strongly fix chondrogenic-enhanced human ASCs at the lesion site of the defective cartilage and extend the survival time of the implanted cells at the defect site so that the cells could differentiate into chondrocytes. The prolonged survival of implanted stem cells, in turn, could exert prolonged paracrine effects and/or engraftment with chondrogenic differentiation.

*Fibrin glue*: Fibrin glue is a two-component topical hemostat and sealant consisting of fibrinogen and thrombin [63]. It is in the market since the late 1970s and is now an FDA-approved tissue sealant for hemostasis, burn wound skin graft attachment and colon sealing [126]. The fibrin, formed after the crosslinking, has structure like that of natural ECM with good biocompatibility, biodegradation and binding capacity to cells and tissue [127]. It is widely used in functional additives delivery and tissue engineering for cell delivery. Clinical study has shown that Fibrin glue with MSCs implantation improves the therapeutic outcome in patients with OA compared with MSCs implantation alone, as graded by the scale from International Cartilage Repair Society grade [128]. However, there is risk of transmitting serological disease from the donors as the components of the Fibrin glue come from the blood of humans or animals.

*Pros and cons*: Nature-based compounds are preferred in biomedical applications due to biocompatibility and biodegradation. Moreover, they come from nature and have certain inherent biological functions, including anti-inflammation, antibacterial activity, antioxidant properties, and the ability to promote cell migration. However, nature-based compounds are often limited by batch-to-batch variability, complex structures, and complicated and costly extraction processes [129]. In addition, these natural compounds usually need modifications to fabricate adhesive hydrogels. It is rarely reported how this modification influences the compound structure of the natural compounds, which is an important consideration as even the molecular weight of the compounds can affect their biological properties [130].

### Synthetic polymers

Synthetic polymers are more biologically inert and have fewer biological functions than natural polymers. However, they have the tunability, various forms, controllable structures, and ease of modifications [131]. Hence, synthetic polymers as engineered adhesive hydrogels for OA therapy are also explored.

*Polyethylene glycol (PEG)*: PEG is a candidate in biomaterials fabrications for tissue engineering applications due to its biocompatibility, non-immunogenicity, and antifouling properties [132]. PEG-based hydrogels are widely explored in tissue adhesives, wound healing and tissue regeneration, including the commercially available Duraseal® and Coseal® [133–135]. Because of the ease of modification through terminal hydroxyl groups, PEG can be modified with adhesive groups including DOPA, acrylate, thiol, NHS-ester, aldehyde, hydrazine and others, some of which have been explored in OA therapy [25, 64, 73, 136–138]. Li et al. [73] synthesized hyper-branched PEG with acrylate groups for tissue adhesion. Later, they used thiol-modified HA to fabricate the adhesive hydrogel and loaded MSCs for cartilage regeneration. Significantly, the MSC-loading adhesive hydrogel repaired full-thickness cartilage defects better than non-treatment and hydrogel alone, providing a promising method for cartilage tissue engineering.

*Poloxamer and poloxamine*: Poloxamer (Pluronic®) and poloxamine (Tetronic®) are amphiphilic blocks of copolymers constituted by polyethylene oxide and polypropylene oxide [139]. The copolymers exhibit thermally induced phase transition in aqueous solutions due to difference in chemical polarities of different blocks [140]. These polymers have been explored in tissue engineering and drug delivery because of their thermoreversible behavior at physiological temperatures and their acceptable biocompatibility and tunability [141]. The precursor solutions of poloxamer or poloxamine are injected at the injury site. Then, the thermally induced phase transition occurs, and the solution becomes a hydrogel. It is easy to flow into irregular places of injured cartilage as it is in solution state when injected. It then forms solid state because of phase transition, resulting in the mechanical interlocking between hydrogel and tissue. As a result, most of these hydrogels show adhesion through mechanical interlocking. These polymers used in OA therapy include Pluronic F127, Pluronic F68, Tetronic 908, etc. [48, 49, 142]. Nascimento et al. [143] developed a sulforaphane-loaded HA-poloxamer hybrid hydrogel for OA therapy. The drug-loaded hydrogel increased type II collagen expression, inhibited proteoglycan consumption, downregulated NF-κB pathway and inhibited PGE2 production in chondrocytes. Generally, mechanical interlocking is relatively weak and other functional groups can be conjugated to these polymers through hydroxyl groups. Lee et al. [144] used thiol groups to modify Pluronic F127 and later fabricated adhesive hydrogel by mixing DOPA-modified HA and thiolated Pluronic F127, which showed excellent tissue adhesion properties.

*Aliphatic polyester*: Aliphatic polyesters are the biodegradable polymers explored in tissue engineering, drug delivery, and medical devices [145]. Aliphatic polyesters include polylactide (PLA), polyglycolide (PGA), polycaprolactone (PCL) and their copolymers. Generally, polyesters have weak tissue adhesion as they are hydrophobic and lack functional groups that form covalent and non-covalent crosslinkings. However, either by physical mixture or by chemical grafting with PEG, these polymers show tissue adhesion through mechanical interlocking induced by phase transition at the physiological temperature. Behrens et al. [146] mixed PLGA with PEG in acetone, which underwent a fibrous mat-to-membrane transition at a temperature of around 31 °C, resulting in mechanical interlocking-induced tissue adhesion. The sealant has higher bursting pressure than that of Fibrin glue. Another choice is to add PEG into polymeric chain during synthesis to fabricate thermo-responsive adhesives for additives release. CircRNA3503 promotes chondrocyte survival by inhibiting apoptosis, modulating cartilage ECM synthesis, and alleviating ECM degradation, showing great promise in preventing OA progression. Tao and co-workers [147] used thermo-responsive poly (D,L-lactide)-*b*-poly(ethylene glycol)-*b*-poly (D,L-lactide) (PDLLA-PEG-PDLLA, PLEL) triblock copolymer-based gels to slowly release circRNA3503-loaded small extracellular vesicles, which protected cartilage and delayed the progression of OA.

*Polyvinyl alcohol (PVA)*: PVA is a synthetic polymer used in industrial, commercial, medical and food applications since the 1930s and is included in the Handbook of Pharmaceutical Excipients [148]. PVA has been reported to be used in multiple biomedical applications such as contact lenses, wound dressings and drug delivery because of its good biocompatibility [149]. Moreover, PVA chain has many hydroxyl groups which give it the potential to form hydrogen bonding with tissue [53]. PVA-based hydrogel can be used as lubricant in OA treatment [54]. It can also be combined with other components to simulate tissue structure. Thangprasert et al. [119] used PVA and gelatin to mimic the structure and function of the natural cartilage tissue. The tissue-mimicking hybrid hydrogel supported the adhesion and proliferation of pre-osteoblast cell line MC3T3-E1 with greater osteogenic density of calcium deposition after mixing with gelatin.

*Pros and cons*: Synthetic polymers have advantages in some studies because of their well-defined structure, strength, and reproducibility. In addition, their chemical inertness enables them to resist chemical breakdowns, increasing the convenience of chemical modification. However, synthetic polymers are mostly biologically inert and additional steps are needed to introduce biological functions in OA therapy.

## Biological functions of the adhesive hydrogels in OA therapy

Inherently, adhesive hydrogels have two functions: to act as functional scaffolds and to be delivery vehicles (Fig. 2c). They can provide highly hydrated microenvironment and mimic native ECM for solutes and nutrient transfer. They also maintain integration between loaded materials and the tissue. Some are even used as lubricants for viscosupplementation. However, due to the high complexity of OA and the complex pathophysiology involved, the introduction of biological functions in OA therapy is important to achieve good therapeutic outcomes. There are two common approaches to introduce biological functions in adhesive hydrogels. The first is fabricating the adhesive hydrogels using polymers or components with intrinsic biological activity such as HA, gelatin, alginate or CS. The second is to add functional additives.

### Functional components

Some polymers used to fabricate adhesive hydrogels have inherent biological functions. These functional components are HA, gelatin, alginate, and CS. Table 1 summarizes examples of these components, respective molecular pathways and pre/clinic state.

**Table 1:** Functional components for biological functions

Functional components	Function	Molecular pathways	Pre/clinic state	References
HA	Anti-inflammation; Pain relief	Combined with TLR-2 and TLR-4→TNF-α, IL-1β, IL-17, MMP-13, iNOS↓	Humans, mice, rats	[150–152]
	Chondrogenesis; Inhibition of degradation; Adaptation to mechanical stress	Combined with ICAM-1→NF-κB↓→IL-6↓ Combined with CD44→PGE2↓, HSP70↑	Guinea pigs, rabbits, mice	[151]
	Promotion of angiogenesis	Combined with CD44→IL-1β↓→MMP 1,2,3,9,13↓	Rabbits, rats, mice	[152–154]
	Improvement of cell proliferation	N/A	Mice	[150–152, 154, 155]
Gelatin	Cell proliferation	N/A	Mice	[155, 156]
Alginate	Adaptation to mechanical stress	N/A	Mice	[96, 97]
	Improvement of cell proliferation	N/A	Mice, horses	[97, 157]
CS	Anti-inflammation; Pain relief; Cell proliferation	p38 MAPK, Erk1/2↓	Rats, humans, rabbits	[158–162]

*CD44* cluster determinant 44, *CS* chondroitin sulfate, *Erk1/2* extracellular signal-regulated kinase 1/2, *HA* hyaluronic acid, *HSP70* heat shock protein 70, *ICAM-1* intercellular adhesion molecule-1, *IL* interleukin, *NF-κB* nuclear factor kappa-B, *iNOS* inducible Nitric oxide synthase, *MMP* matrix metalloproteinase, *p38 MAPK* p38 mitogen-activated protein kinase, *PGE2* prostaglandin E2, *TLR* toll-like receptor, *TNF-α* tumor necrosis factor α, *N/A* not applicable

The amount of HA is often lower in synovial fluid of osteoarthritic joints than in healthy joints. Hence, intra-articular injection of HA is an FDA-approved method to treat OA for enhancing lubrication and reverse the proinflammatory pathways [153]. In OA therapy, HA reduces the production of pro-inflammatory cytokines and SASP factors like interleukin (IL)-1β and IL-6, as well as tumor necrosis factor α (TNF-α); it suppresses MMPs and PGE2 syntheses via CD44 receptor. HA also downregulates p65 NF-κB and IκBα phosphorylation activated by LPS via intercellular adhesion molecule-1 receptor [150]. It was shown that HA could effectively maintain the chondrogenic phenotype in pig model and change the trabecular structure of subchondral bone in rod-like way, reducing cartilage loading during mechanical impact [163, 164]. HA also facilitates cell migration and angiogenesis [153], thus promoting tissue regeneration [165] in dose-dependent manner. As a result, using HA to fabricate adhesive hydrogels introduces inherent biological functions [166].

Gelatin is native to ECM. It is a product of collagen hydrolysis and is less immunogenic than collagen [167]. Besides, it has instructive signals, including the arginine-glycine-aspartic acid sequence, promoting cell adhesion, proliferation, and differentiation [168]. In OA therapy, recent studies indicated that gelatin supports chondrogenesis as shown by increased staining of chondrogenic lineage differentiation of bone marrow MSCs cultured on gelatin [169, 170].

Alginate is a nature-based polymer promoting the mineralization of ECM in vitro [95]. Igarashi et al. [171] showed that alginate (1000 kDa) had the potential for OA prevention and treatment by reducing the joint friction coefficient and alleviating articular cartilage degeneration. Animal experiments demonstrated that alginate-gelatin scaffolds had excellent mechanical and relaxation properties which provided favorable physical environment for ECM remodeling [74], and inducing cartilage differentiation [97, 172, 173].

CS is another component of ECM that exerts biological functions essential to meniscus microstructure and mechanical properties. Downregulation of GAG content and collagen fibre tissue disrupts the impact loading and collagen sliding [174], resulting in OA [175]. Moreover, the energy metabolism of chondrocytes in OA switches from oxidative phosphorylation to anaerobic glycolysis under the imposition of nutrient stress [40], inhibiting 5'-AMPK signaling and increasing the pro-catabolic responses to IL-1β and TNF-α in chondrocytes [176]. Furthermore, chronic hyperglycemia induces overproduction of advanced-glycation end products in joint tissue, accelerating the formation of joint contracture [177]. Based on the above-mentioned background, highly purified CS can decrease p38 MAPK and signal-regulated kinase 1/2 phosphorylation stimulated by IL-1β, NF-κB [158], TNF-α, COX-2 and iNOS [159]. The inflammation is then reduced due to metabolic and mechanical disorders, preventing the progression of OA. The positive effects of CS in 3D fibrin-alginate hydrogels on cartilage matrix production and chondrocyte proliferation have been demonstrated in pig models [178].

### Functional additives

**Fig. 3:**
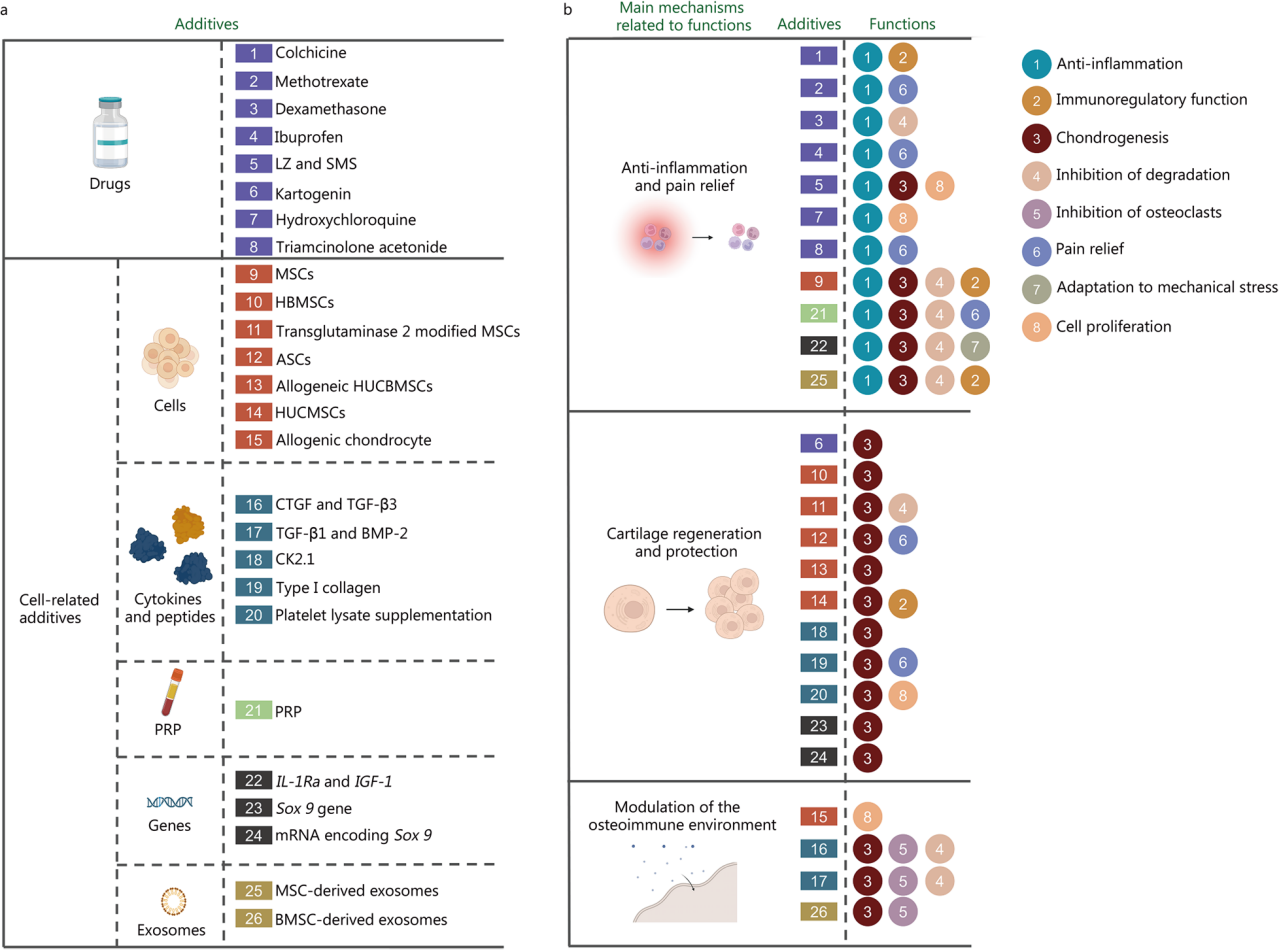
Functional additives-based treatments and main biological mechanisms involved in OA treatment. **a** Common functional additives in OA treatment. These additives contain drugs, and cell-related additives, including cells, cytokines and peptides, PRP, genes and exosomes. **b** The main biological mechanisms of additives and their functions in OA therapy. The biological mechanisms include: 1) anti-inflammation and pain relief; 2) cartilage regeneration and protection; 3) modulation of osteoimmune environment. The functions include: 1) anti-inflammation; 2) immunoregulatory function; 3) chondrogenesis; 4) inhibition of degradation; 5) inhibition of osteoclasts; 6) pain relief; 7) adaption to mechanical stress; 8) cell proliferation. OA osteoarthritis, LZ and SMS Chinese medicine Lingzhi and San-Miao-San, MSCs mesenchymal stem cells, HBMSCs human bone mesenchymal stem cells, ASCs adipose stem cells, HUCBMSCs human umbilical cord blood derived mesenchymal stem cells, HUCMSCs human umbilical cord mesenchymal stem cells, CTGF connective tissue growth factor, TGF-β transforming growth factor-β, PRP platelet-rich plasma, IL-1Ra interleukin-1 receptor antagonist, IGF-1 insulin-like growth factor-1, Sox 9 SRY-related high mobility group-box 9, BMSC bone mesenchymal stem cell. It was created utilizing the templates on BioRender.com as a reference

In addition to the components used in fabricating adhesive hydrogels, functional additives can also be added to the adhesive hydrogels for introducing various biological functions. These additives which have been and potentially can be loaded into adhesive hydrogels are summarized with their functions (Fig. 3; Table 2). It is worth noting that these additives can be used alone or together, and herein, they are discussed separately for clarity. They can be categorized as: drugs and cell-related additives.

**Table 2:** Functional additives for biological functions

Categories	Name	Function	Pre/clinic State	Hydrogel	References
Drugs	Colchicine	Anti-inflammation; Immunoregulatory function	Rats	Chitosan	[179]
	Methotrexate	Pain relief; Anti-inflammation	Rats	HA	[180]
	Dexamethasone	Chondrogenesis; Anti-inflammation; Inhibition of ECM degradation	Rats	HA	[181]
	Ibuprofen	Pain relief; Anti-inflammation	Mice Rats	Carbopol® 934; PLGA/gelatin/PVA	[182, 183]
	Chinese medicine Lingzhi and San-Miao-San	Differentiation of osteogenic; Anti-inflammation; Chondrogenesis	Rats	HA	[184]
	Kartogenin	Chondrogenesis; Cell proliferation	Rabbits Rats	PLGA-PEG-PLGA; Gelatin	[185, 186]
	Hydroxychloroquine	Anti-inflammation; Cell proliferation	Mice	MMP-13/pH-responsive ferritin nanocages (CMFn)	[187]
	Triamcinolone acetonide	Anti-inflammation; Pain relief (some scholars do not recommend it)	Rats Human	PLA/methoxy-PEG-PDL None	[188, 189]
	Simvastatin	Chondrogenesis; Anti-inflammation	Mice	Gelatin	[156]
	Chitooligosaccharide-salicylic acid conjugate	Antioxidation	Mice	Alginate, Gelatin	[190]
	Eicosapentaenoic acid	Immunoregulatory function; Chondrogenesis	Mice	Gelatin	[191]
Cells	MSCs	Chondrogenesis; Inhibition of ECM degradation	Rats	HA	[192]
	Human bone mesenchymal stem cells	Chondrogenesis	Rat	Gelatin	[160]
	Membrane-modified MSCs by transglutaminase 2	Chondrogenesis; Inhibition of ECM degradation	Rabbits	N/A	[193]
	Adipose-derived stem cells	Chondrogenesis; Pain relief	Human Rabbits	None HA-PNIPAAm-CL	[194, 195]
	Allogeneic human umbilical cord blood derived MSCs	Chondrogenesis	Human	HA	[196]
	Human umbilical cord mesenchymal stem cells	Chondrogenesis; Immunoregulatory function	Pigs	HA	[197]
	Allogenic chondrocytes	Chondrogenesis; Inhibition of ECM degradation; Cell proliferation	Human Rabbits	Type I collagen Chitosan	[198, 199]
Cytokines and Peptides	TGF-β1, BMP-2	Chondrogenesis; Inhibition of ECM degradation; Inhibition of osteoclasts	Rabbits; Rat	PCEC	[200, 201]
	CTGF, TGF-β3	Chondrogenesis; Inhibition of ECM degradation; Inhibition of osteoclasts	Rabbits	PLGA	[202]
	Biphasic CK2.1 (QIKIWFQNRRKWKKMVPSDPSYEDMGGC, 95%)	Chondrogenesis	Mice	β-glycerophosphate chitosan	[203]
	Type I collagen	Chondrogenesis	Cells	Sodium alginate	[204]
	Platelet lysate supplementation	Chondrogenesis; Cell proliferation	Cells	Dextran-tyramine	[205]
PRP	PRP	Pain relief; Chondrogenesis; Inhibition of ECM degradation	Rabbits, Human	PRP	[69, 206]
Genes	*IL-1Ra* and *IGF-1* gene	Inhibition of ECM degradation; Anti-inflammation; Adaptation to mechanical stress	Rabbits	Chitosan	[207]
	*Sox 9* gene	Chondrogenesis	Mice	PEO and PPO	[208, 209]
	mRNA encoding *Sox 9*	Chondrogenesis	Cells	Gene-activated matrixes (GAM)	[210]
Exosome	MSC-derived exosomes	Anti-inflammation; Chondrogenesis; Immunoregulatory function	Rabbits Rats	HA-NB/Gelatin None	[211] [212]
	BMSC-derived exosomes	Chondrogenesis; Inhibition of osteoclasts; Cell proliferation	Rats	Alginate, chondroitin sulfate and silk fibroin	[74]

*BMSC* bone mesenchymal stem cell, *BMP-2* bone morphogenetic protein-2, *CS* chondroitin sulfate, *CTGF* connective tissue growth factor, *ECM* extracellular matrix, *HA* hyaluronic acid, *HA-NB* o-nitrobenzyl alcohol-modified HA, *IGF*-1 insulin-like growth factor-1, *IL-1Ra* interleukin-1 receptor antagonist, *MSCs* mesenchymal stem cells, *PCEC* poly(ε-caprolactone)-poly(ethyleneglycol)-poly(ε-caprolactone), *PDL* poly(δ-decalactone), *PEG* polyethylene glycol, *PEO* poly(ethylene oxide), *PLA* polylactide, *PLGA* polylactide-*co*-glycolide, *PPO* poly(propylene oxide), *PRP* platelet-rich plasma, *PVA* polyvinyl alcohol, *Sox 9* SRY-related high mobility group-box 9, *TGF-β* transforming growth factor-β, *N/A* not applicable

#### Drugs

Non-steroidal anti-inflammatory drugs (NSAIDs) and corticosteroids are the two major drugs of OA treatment, which reduce inflammation and relieve pain (Fig. 3). However, oral NSAIDs increase the incidence of gastrointestinal disorders [213]. Corticosteroids are known to bring adverse effects such as infection and bone loss [214]. Other inflammation-modulating drugs like methotrexate, hydroxychloroquine and DEX, are also effective in OA animal models [180, 181, 187], which reduce joint swelling and inhibit OA progress.

*Pros and cons*: Drugs are generally applied for symptom management. Few can reverse OA pathologic progress. Furthermore, powerful side effects like non-selectivity, chondronecrosis and infection make repeated injections inappropriate. If the drugs could remain in the joint over a longer time, beneficial effects could be maximized and systemic adverse effects minimized. As a result, carriers like adhesive hydrogels will be needed.

#### Cell-related additives

*Cells*: Cell therapy is among the most promising techniques for repairing damaged or destroyed tissue [215]. Chondrocytes and stem cells are the most used cells for getting injected in the joints. The former is direct supplementation for cartilage injury, and latter can be induced to differentiate into bone cells and chondrocytes in specific situation. MSCs reduce local inflammation, prevent chondrocyte hypertrophy and apoptosis, and differentiate into chondrocytes that form cartilage [192]. MSCs also induce macrophage polarization to M2 phenotypes and increase the secretion of IL-10, thereby inhibiting inflammation [73]. It was reported that intra-articular injection of autologous MSCs provided pain relief to patients with knee OA [192, 194].

*Cytokines and peptides*: As the two main cytokines used for tissue regeneration for decades, TGF-β and fibroblast growth factor have been injected to stimulate native chondrocyte proliferation or chondrogenic differentiation of resident progenitor or stem cells [216, 217]. Zhou et al. [200] fabricated injectable and thermos-responsive hydrogel to load TGF-β for cartilage repair. They found that the system promoted full-thickness defect regeneration on rat knees. In osteonecrotic OA rabbit model of hip joint, basic fibroblast growth factor-loaded gelatin showed improved Mankin scoring (degree of articular cartilage degeneration) by promoting OA repair [218]. In rabbits, the short-term release of connective tissue growth factor recruited synovial MSCs to the loss site and formed an integrated fibre matrix with continuously released TGF-β. Moreover, it remodeled the fibrous matrix into fibrocartilage matrix, repaired meniscus tissue and improved its function [202]. Other anti-inflammatory cytokines, for example, interleukin IL-4, IL-10, and IL-13, have also been loaded to hydrogels to treat OA [219].

A few peptides were found to have the ability to induce chondrogenesis without inducing chondrocyte hypertrophy, which can be used for cartilage repair in OA. CK2.1, a mimetic peptide of bone morphogenetic protein receptor, is one of these peptides with the potential to induce ECM secretion and chondrogenesis without the induction of hypertrophy [203]. LL37 is a peptide known for antimicrobial function, immune modulation, and the ability to promote bone healing by MSC recruitment [220]. Liu et al. [221] used a composite scaffold to load CK2.1 at upper layer for cartilage regeneration and LL37 at bottom layer for bone regeneration. The composite scaffold enhanced the repair of cartilage and subchondral bone defect, offering a novel therapeutic strategy for patients with articular osteochondral defect. In mice model, biphasic system of CK2.1 peptide-coated β-glycerophosphate/chitosan and LL37-modified layered double hydroxide/chitosan induced cartilage formation without provoking chondrocyte hypertrophy. This may be one of few peptides developed or proteins found with this ability and can be used for cartilage repair in OA related cartilage loss [203, 221].

*Platelet-rich plasma (PRP)*: PRP is a type of concentrated platelets, isolated by centrifuging autologous whole blood [222]. PRP contains hundreds of cytokines, adhesive proteins, small molecules, ions and abundant autologous growth factors. In the joint, intra-articular injection of PRP affects local and infiltrating cells. It stimulates cartilage formation and improves the symptoms of knee OA by regulating the microenvironment, cell composition and proliferation [69, 206]. Besides, PRP has anti-inflammatory properties through its effects on canonical NF-κB signaling pathway in chondrocytes and macrophages [223]. Its components, like TGF-β and PDGF, interact with cells involved in immune response and angiogenesis and regulate ECM’s microenvironment. It is, therefore, a popular candidate as a functional additive for OA therapy [69].

*Genes*: Gene therapy is the term for delivering nucleic acids to the tissue of interest by viral [224] and non-viral vectors [225]. Combining gene transfer with hydrogels may provide promising tools for human tissue engineering and regenerative medicine strategies [49]. Transcription factor Sox 9 enhances differentiation of chondrocytes [208, 209]. In vitro study showed that mRNA encoding Sox 9 strongly induced synthetic cartilage and the expression of muscle-specific markers [210]. Madry et al. [27] prepared thermosensitive hydrogel-based on PEO-PPO–PEO poloxamers. The hydrogels controllably released therapeutic (Sox 9) rAAV vectors to improve the repair of full-thickness chondral defects in minipig.

*Exosomes*: Exosomes are natural membrane-bound nanocarriers that contain diverse biomolecules such as proteins, lipids, and nucleic acids [226]. Exosomes are derived from various cells through exocytosis and transfer biological signals between local or distant cells, exhibiting a variety of biological regeneration functions [227]. The exosomes derived from MSC inhibit immune response, and enhance cartilage differentiation of progenitor cells and cartilage tissue regeneration, which can be delivered by hydrogels [228]. They are an effective alternative treatment for OA in osteochondral tissue [13, 212, 229]. A report showed that BMSC-derived exosomes, loaded in DOPA-modified alginate, CS, and regenerated silk fibroin adhesive hydrogel, could recruit BMSCs to migrate and expand. This promoted proliferation and differentiation of BMSC, accelerating the regeneration of in situ cartilage defects, and reshaping ECM [74]. Many studies have proved the effectiveness and feasibility of MSC-derived exosomes in OA therapy. However, there is a lack of consensus on the best method for obtaining high yields of pure exosomes, in addition to the cumbersome purification process, which adversely affects the potential of clinical translation [230].

*Pros and cons*: Compared with drugs, cell-related additives have more biological functions that promote tissue regeneration and reverse the OA progression. However, none of these additives performs up to the mark. Cells suffer from low viability during injection, poor cell targeting, and unsatisfactory stem cell differentiation. PRP has large batch-to-batch variations in preparation which compromises the reproducibility. For gene therapy, safety and transfection efficiency require improvements. For exosomes, the lack of best method for obtaining high yields of pure exosomes and a cumbersome purification process reduces the therapeutic effects of exosomes.

## Perspective and outlook

OA is a degenerative disease and one of the leading causes of disability worldwide. Trauma, age, genetics, inflammation, metabolic dysfunction, occupational factors and unhealthy living habits are related to the occurrence and development of OA. Tissue engineering and drug administration are among the most promising therapeutic strategies for OA management where hydrogels play a pivotal role. Tissue engineering-based approaches have flourished in the last decade to fulfil all the needs for treating OA. Hence, the development of cell-free scaffolds like MaioRegen [231] or Trufit [232] have been clinically tested in articular cartilage repair with promising outcomes that can be applied in OA management. As a scaffold in tissue engineering, hydrogels provide physical support to cells while being compatible and biodegradable with porous 3D structure [233]. Although very promising, there are still a limited number of clinical products in OA therapy, like CaReS® [234], BioSeed® [235] and Hyalograft® C [236]. In addition to cell viability, scaffold biomechanics, and the method for implantation, another issue is how to achieve stable or proper tissue adhesion and integration between the scaffolds and the cartilage tissue.

Topical or oral NSAIDs are the common approaches for managing pain and discomfort during the early stages of OA [3]. When the disease progresses, a more invasive therapy is needed, and the intra-articular injection of corticosteroids or viscosupplements is used to relieve the patient from inflammation and pain [237]. However, as discussed, these additives have short half-life, and are eliminated from synovial fluid in less than 4 h. This leads to poor bioavailability and the need for higher doses with undesired side effects [238]. Hence, drug delivery systems overcoming the weakness of the free drug are of great interest and, and for this purpose, hydrogels, micelles or polymeric particles have been tested in clinical trials including liposome-based [239–241], PLGA-based [242], and HA-based [243–245] systems.

Adhesive hydrogels are promising candidates in OA therapy as they possess cartilage tissue-like properties with inherent adhesiveness. These hydrogels are used as tissue scaffolds, functional additive carriers, and lubricants. Some of the hydrogels in clinical trials exhibit adhesion to tissue, like the polyacrylamide-based ones (Table 3).

**Table 3:** Current clinical trials using hydrogels as potential therapies for the management of OA (Data from: https://clinicaltrials.gov)

Study title	Material	Groups	Participants	Primary outcome	Phase	Status	Identifier
HUPS: hyalgan use in painful shoulder	Sodium hyaluronate	PBS control; 20 mg sodium hyaluronate injection	602	Improvement in shoulder pain on movement compared to the PBS control group	3	Completed	NCT00377624
Intra-articular PVA hydrogel in knee OA	PVA Hydrogel; Hylan G-F20 (Synvisc-One®)	2 ml injection of PVA hydrogel; 6 ml injection of Hylan G-F20 (Synvisc-One®)	43	To test if there are any adverse events at the injection site	N/A	Completed	NCT04693104
Intra-articular polyacrylamide hydrogel in knee OA	Polyacrylamide hydrogel with silver ions “Argiform”	Hydrous biopolymer with silver ions “Argiform”; Saline	144	Change of the total WOMAC score (WOMAC-T) in grade II-III OA patients	N/A	Unknown	NCT03897686
New hydroxyethyl cellulose hydrogel for the treatment of the pain of knee arthrosis	Hydroxyethyl cellulose hydrogel	Hydrogel injection	50	Pain assessment in terms of percentage of pain reduction using a visual analog scale	N/A	Recruiting	NCT04061733
Treatment of knee OA with PAAG-OA (ROSA)	Polyacrylamide hydrogel (PAAG-OA)	Intra-articular injection of 6 ml PAAG-OA; Intra-articular injection of 6 ml Synvisc-One® (HA)	238	Comparing one injection of PAAG-OA with one injection of Synvisc-One® on pain over 6 months in subjects with knee OA	N/A	Active, not recruiting	NCT04045431
Safety and effectiveness study of a non-crosslinked HA Alkylamide HYADD(TM) 4 hydrogel for OA of the knee	Non-crosslinked HA Alkylamide	HYADD (TM) 4 hydrogel intra-articular injection; Placebo intra- articular injection	332	WOMAC pain sub-score	N/A	Unknown	NCT02187549
Hymovis™ versus placebo in knee OA (Hymovis)	Non-crosslinked HA Alkylamide (Hymovis)	Hymovis intra-articular injection; Phosphate buffered saline injection	800	WOMAC pain sub-score	3	Completed	NCT01372475
PAAG-OA treatment for knee OA (IDA)	Polyacrylamide hydrogel	Intra-articular polyacrylamide hydrogel injection	49	WOMAC pain sub-score	N/A	Active, not recruiting	NCT04179552
Arthrosamid injection for OA knee	Crosslinked polyacrylamide (Arthrosamid)	Intra-articular injection of water and crosslinked polyacrylamide	60	Changes in the WOMAC score between baseline (pre-injection) and 6 months and 12 months post-injection	N/A	Not yet recruiting	NCT05086068
Aquamid reconstruction for OA of the knee	Polyacrylamide hydrogel (Aquamid)	Intra-articular injection of 3 ml aquamid reconstruction (AR) to the knee	50	Change from baseline in the pain sub-score of the WOMAC	N/A	Unknown	NCT03067090
Evaluation of the evolution of biological and imaging markers of bone and cartilage degradation in patients with knee OA receiving intra-articular injections of a hyaluronan derivative HYMOVIS®	HYMOVIS (obtained by hydration of the HA-based derivative named HYADD4p5)	Two treatment cycles of two injections of HYMOVIS® at baseline and 6 months	50	Assess the variation of type II collagen-specific biomarkers (Coll2-1, Coll2-1NO2 & CTX-II) after HYMOVIS® treatment versus baseline	N/A	Completed	NCT04293861
Geniculate artery embolization for treatment of OA	Embozene™ Color-Advanced hydrogel microspheres coated with an inorganic perfluorinated polymer used for embolization	Transcatheter arterial embolization using Embozene™	23	Change in knee pain using WOMAC score	N/A	Recruiting	NCT04379700
To look at the characteristics of synovial fluid and cartilage matrix in the osteoarthritic knee after HA injection	EUFLEXXA® is a hyaluronate hydrogel produced from bacteria	EUFLEXXA® intra-articular injection	12	To identify imaging markers for characterizing the biochemical profiles in synovial fluid and cartilage in knee OA 3 months after HA injection	4	Completed	NCT01895959
Efficacy and safety of Hymovis ONE® (32 mg/4 ml) intra-articular injection in active patients with knee overuse syndrome	Hymovis® ONE	Hymovis® ONE (32 mg/4 ml) intra-articular mono injection.	31	The efficacy of Hymovis® ONE (32 mg/4 ml) single intra-articular injection in the management of pain caused by knee OA due to overuse: KOOS questionnaire	N/A	Completed	NCT04661111

Western Ontario and McMaster Universities Arthritis Index (WOMAC) consists of three subscales: pain (five questions, 0–20 points), stiffness (two questions, 0–8 points), and physical function (17 questions, 0–68points). Higher scores represent worse pain, stiffness, and functional limitations. *Coll2* type II collagen, *Coll2-1NO2* nitrate form of Coll2-1, *CTX-II* c-terminal crosslinking telopeptide of type-II collagen, *OA* osteoarthritis, *PVA* polyvinyl alcohol, *KOOS* knee injury and osteoarthritis outcome score, *N/A* not applicable

As discussed in this manuscript, adhesive hydrogels improve therapeutic outcomes through offering stable integration between tissue and implants. However, achieving stable and strong integration in a highly humid environment remains a challenge. Traditional OA adhesive hydrogels, with mechanical interlocking-induced adhesion, ‘passively’ rely on the state of cartilage tissue. Therefore, some newly developed ‘positive interlocking methods’, including the gecko-inspired and micro-needle-based adhesion, can be excellent alternatives for more stable integration. Inspired by endoparasite *Pomphorhynchus laevis*, Yang et al. [246] developed biphasic microneedle array that mechanically interlocked with tissue through swellable microneedle tips. The needles are inserted to tissue in dry state, and they swell upon contact with body fluids to offer mechanical interlocking. However, nearly all mechanical interlockings, including gecko and microneedle-based, need irregular surfaces or soft structures. Because cartilage tissue is relatively hard and firm, mechanical interlocking may result in limited adhesion. A combination of mechanical interlocking and intermolecular interaction can be another solution. Ma et al. [247] created a gecko-like adhesive and added a polymer coating containing catechol groups to achieve high underwater adhesion strength.

Intermolecular interactions integrate at molecule level. The double-network-based strategy is one of the strongest interactions. Double-network hydrogels are composed of two networks with contrasting structures, which can promote elasticity and stiffness. They are needed because single-network gels are either too brittle or too soft [248, 249]. This strategy achieves higher adhesion by introducing an energy dissipation network in cohesion design. Furthermore, due to energy dissipation, such adhesives have high bulk strength, making them mechanically robust to withstand significant compressions [250, 251].

However, because double-network-based adhesive hydrogels are frequently pre-made, site delivery may be challenging. Open surgery may be required to place the materials, limiting their clinical applications. They are, therefore, not appropriate for translation from standpoints of production, patients, and clinicians. Given the strength of ‘Double-network-based technique’, one-step process for fabricating double-network hydrogel, with physical interactions created in a physiological environment, may broaden the applicability of traditional double-network adhesive in OA therapy [252, 253]. This also reminds us that ease of use must be considered when developing adhesive hydrogels.

When designing adhesive hydrogels for OA therapy, a ‘tuneable approach’ is suggested, recognizing these adhesive hydrogels’ functions and the OA’s pathogenic state. For example, for lubricants or drug delivery applications, the adhesive hydrogels need stable adhesion under the stress caused by joint movement. However, for adhesive hydrogels that mechanically support the joints, especially in critically injured cartilage, higher bulk strength and high adhesion strength are required. The concept of living glues produced through bacterial engineering offers a good way to design tuneable bioadhesives. Zhang et al. [254] used *Bacillus subtilis* with genetic engineering and materials science to generate tuneable living glues. The engineered *Bacillus subtilis* biofilms had adhesive components from three marine systems including barnacle, mussel and sandcastle worm. By inducible enzymatic modification, these adhesives show tuneable adhesion performance. However, as OA is a highly inflammatory environment, avoiding immune response caused by residual bacteria or bacterial secretions in living adhesives may be a problem to overcome before this technique can impact OA therapy.

As OA is a disease with highly complicated pathogenesis and multiple molecular pathways, introducing biological functions to the adhesive hydrogels will accelerate the tissue healing. Over recent years, increasing evidence has shown that OA is closely associated with an innate immune system, which makes immunomodulation important in OA therapy. Currently, there are three immunomodulatory strategies: autologous or allogenic cell delivery, genetic engineering or gene therapy for resident and exogenous cell population modulation, and biomaterial-based immunomodulation [255]. Stems cells, biomaterials, genes, and cytokines are reported to function as immunomodulators. Thus, from immunomodulation aspect, containing those functional additives is a good option for the OA-targeting adhesive hydrogels systems. According to clinicaltrial.gov website, there are 179 clinical trials testing MSCs in OA therapy, 133 using PRP, more than 60 testing intra-articular injection of corticosteroids, 24 involving gene therapy and 1 using exosomes. All these bioactive entities provide characteristics for improving OA conditions. Many, however, deal with a short half-life and easy clearance from the intended site, leading to low bioavailability. Thus, the combination of adhesive hydrogels and those functional additives can not only incorporate biofunctions to the adhesives but also overcome this major limitation of the existing therapies used in the clinic.

Diverse functional additives can be used for complexation to improve therapeutic outcomes. It has been reported that including TGF-β1/DEX/celecoxib together promotes cartilage formation of human MSCs in vitro and reduces OA symptoms of articular cartilage in animal models [256]. Additionally, the full articular cartilage defects repaired by TGF-β1/DEX/celecoxib complex are resurfaced by neo-tissue with similar thickness, cell arrangement, and color to the normal neighboring cartilage and abundant GAG after 12 weeks [65, 257].

Recent advances in high throughput analysis, such as genomics, proteomics and glycomics, can assist in understanding the molecular basis of OA pathogenesis and effects of current treatments. Additionally, clinical studies comparing the efficacy of the new approaches with traditional ones would, in the long-term, greatly enhance the advances in OA management. Moreover, studies on action mechanisms of the current adhesive hydrogel-based treatments will likely enable the development of more sophisticated therapies. While information from mechanistic aspects is lacking, it has been reported that adhesive hydrogels help in pain management and improve the patient’s life quality [15]. Indeed, only two current clinical trials have focused primarily on cartilage and/or wound healing biomarkers (Table 3). Besides, there is a need for study on adhesive hydrogels about how different adhesion mechanisms and components influence the OA microenvironment. For example, the oxidation of phenol-modified polymers has traditionally been considered to provide tissue adhesion to soft tissue [258–260]. However, as oxidative stress is closely associated with inflammation, the influence of the oxidants used during adhesive hydrogel formation should be thoroughly investigated. This parameter of tissue responses should be one of the key factors that define adhesive hydrogels for OA, as with adhesive strength, bursting pressure, swelling ratio and degradation properties.

Material science does not address the metabolic disorders caused by hypoxia and metabolic syndrome which contribute to the progression of OA [261, 262]. The hypoxia-inducible factors-2α expression causes OA by promoting Fas-mediated chondrocyte apoptosis [261]. Induced by chronic excess of glucolipid metabolism, synovium secretes adipokines, such as free fatty acids, leptin, and adiponectin, which increase the expression of cartilage-degeneration-related genes in chondrocytes [262, 263]. Reseland et al. [264] reported that leptin is released upon local mechanostimulation, which might be associated with osteoblastic development in subchondral bone remodeling. These findings can offer new pathways for OA therapy.

## Conclusions

The functions the future adhesive hydrogel-based treatment must fulfil are: 1) tuneable adhesion between implants and cartilage tissue according to various conditions, aiming at offering rigid integration for mechanical stability and additives delivery; 2) biological functions achieved by functional additives and/or the functional components. While most current research attempts to meet these expectations, developing more clinically oriented functional adhesive hydrogels in OA treatment needs further work. Multiple pathways are involved in OA, and current state of the art in adhesive hydrogel development is far away from true biological functional replacement (Fig. 1). A multimodal approach is needed to achieve breakthroughs in OA therapy, adhesive hydrogel development for clinically successful OA therapy requires a highly interdisciplinary framework that includes disciplines of chemistry, pharmaceutics, biology, and clinical medicine.

## Data Availability

Not applicable.

## Abbreviations

AKT: Serine/threonine kinase Akt, also known as protein kinase B
AMPK: Adenosine 5’-monophosphate (AMP)-activated protein kinase
ASCs: Adipose stem cells
BMMC: Bone marrow mononuclear cell
BMSC: Bone mesenchymal stem cell
BMP-2: Bone morphogenetic protein-2
CD44: Cluster determinant 44
CS: Chondroitin sulfate
CTGF: Connective tissue growth factor
DOPA: Dopamine
DEX: Dexamethasone
ECM: Extracellular matrix
GAG: Glycosaminoglycan
GelMA: Methacrylated Gelatin
HA: Hyaluronic acid
HA-NB: O-nitrobenzyl alcohol-modified HA
HBMSC: Human bone mesenchymal stem cell
HMSC: Human mesenchymal stem cell
HSP70: Heat shock protein 70
HUCMSC: Human umbilical cord mesenchymal stem cell
IGF-1: Insulin-like growth factor-1
IL: Interleukin
IL-1Ra: Interleukin-1 receptor antagonist
iNOS: Inducible nitric oxide synthase
LZ and SMS: Chinese medicine Lingzhi and San-Miao-San
MAP: Mussel adhesive proteins
MAPK: Mitogen-activated protein kinase
MMP: Matrix metalloproteinase
MSC: Mesenchymal stem cells
mTORC1: Mechanistic target of rapamycin complex 1
NF-κB: Nuclear factor kappa-B
NSAIDs: Non-steroidal anti-inflammatory drugs
N/A: Not applicable
OA: Osteoarthritis
PCEC: Poly(ε-caprolactone)-poly(ethyleneglycol)-poly(ε-caprolactone)
PCL: Polycaprolactone
PDL: Poly(δ-decalactone)
PEG: Polyethylene glycol
PEO: Poly(ethylene oxide)
PGA: Polyglycolide
PGE2: Prostaglandin E2
PLA: Polylactide
PLGA: Polylactide-*co*-glycolide
PPO: Poly(propylene oxide)
PRP: Platelet-rich plasma
PVA: Polyvinyl alcohol
p38 MAPK: p38 mitogen-activated protein kinase
rAAV: Recombinant adeno-associated virus vector
RANKL: Receptor activator of NF-κB ligand
SASP: Senescence-associated secretory phenotype
Sox 9: SRY-related high mobility group-box 9
TGF-β: Transforming growth factor-β
TLR: Toll-like receptor
TNF-α: Tumor necrosis factor-α

## References

[1] Nelson AE (2018). Osteoarthritis year in review 2017: clinical. Osteoarthr Cartil.

[2] Hunter DJ, March L, Chew M (2020). Osteoarthritis in 2020 and beyond: a lancet commission. Lancet.

[3] Quicke JG, Conaghan PG, Corp N, Peat G (2022). Osteoarthritis year in review 2021: epidemiology & therapy. Osteoarthritis Cartilage.

[4] Loeser RF (2017). The role of aging in the development of osteoarthritis. Trans Am Clin Climatol Assoc.

[5] Allen KD, Thoma LM, Golightly YM (2022). Epidemiology of osteoarthritis. Osteoarthritis Cartilage.

[6] Ma L, Zheng X, Lin R, Sun AR, Song J, Ye Z (2022). Knee osteoarthritis therapy: recent advances in intra-articular drug delivery systems. Drug Des Devel Ther.

[7] Steinmeyer J, Bock F, Stöve J, Jerosch J, Flechtenmacher J (2018). Pharmacological treatment of knee osteoarthritis: special considerations of the new german guideline. Orthop Rev (Pavia).

[8] Grässel S, Muschter D (2020). Recent advances in the treatment of osteoarthritis. F1000Res.

[9] Zhang Z, Schon L (2022). The current status of clinical trials on biologics for cartilage repair and osteoarthritis treatment: an analysis of ClinicalTrials.gov data. Cartilage.

[10] Arroll B, Goodyear-Smith F (2004). Corticosteroid injections for osteoarthritis of the knee: meta-analysis. BMJ.

[11] Bae DK, Song SJ, Yoon KH, Heo DB, Kim TJ (2013). Survival analysis of microfracture in the osteoarthritic knee-minimum 10-year follow-up. Arthroscopy.

[12] Wakitani S, Imoto K, Yamamoto T, Saito M, Murata N, Yoneda M (2002). Human autologous culture expanded bone marrow mesenchymal cell transplantation for repair of cartilage defects in osteoarthritic knees. Osteoarthritis Cartilage.

[13] Kim YG, Choi J, Kim K (2020). Mesenchymal stem cell-derived exosomes for effective cartilage tissue repair and treatment of osteoarthritis. Biotechnol J.

[14] Chiang CW, Hsiao YC, Jheng PR, Chen CH, Manga YB, Lekha R (2021). Strontium ranelate-laden near-infrared photothermal-inspired methylcellulose hydrogel for arthritis treatment. Mater Sci Eng C Mater Biol Appl.

[15] Wei W, Ma Y, Yao X, Zhou W, Wang X, Li C (2020). Advanced hydrogels for the repair of cartilage defects and regeneration. Bioact Mater.

[16] Wang S, Qiu Y, Qu L, Wang Q, Zhou Q (2022). Hydrogels for treatment of different degrees of osteoarthritis. Front Bioeng Biotechnol.

[17] Jeon HY, Shin EY, Choi JH, Song JE, Reis RL, Khang G (2018). Evaluation of saponin loaded gellan gum hydrogel scaffold for cartilage regeneration. Macromol Res.

[18] Holland TA, Bodde EW, Cuijpers VM, Baggett LS, Tabata Y, Mikos AG (2007). Degradable hydrogel scaffolds for in vivo delivery of single and dual growth factors in cartilage repair. Osteoarthritis Cartilage.

[19] Xue X, Hu Y, Deng Y, Su J (2021). Recent advances in design of functional biocompatible hydrogels for bone tissue engineering. Adv Funct Mater.

[20] Lim KS, Abinzano F, Bernal PN, Albillos Sanchez A, Atienza-Roca P, Otto IA (2020). One-step photoactivation of a dual-functionalized bioink as cell carrier and cartilage-binding glue for chondral regeneration. Adv Healthc Mater.

[21] Rethi L, Lu L, Huynh VT, Manga YB, Rethi L, Mutalik C (2021). Bioactive glass fiber-reinforced plastic composites prompt a crystallographic lophelia atoll-like skeletal microarchitecture actuating periosteal cambium. ACS Appl Mater Interfaces.

[22] Chen J, Yang J, Wang L, Zhang X, Heng BC, Wang DA (2020). Modified hyaluronic acid hydrogels with chemical groups that facilitate adhesion to host tissues enhance cartilage regeneration. Bioact Mater.

[23] Bu Y, Pandit A (2022). Cohesion mechanisms for bioadhesives. Bioact Mater.

[24] Duan W, Bian X, Bu Y (2021). Applications of bioadhesives: a mini review. Front Bioeng Biotechnol.

[25] Li F, Truong VX, Fisch P, Levinson C, Glattauer V, Zenobi-Wong M (2018). Cartilage tissue formation through assembly of microgels containing mesenchymal stem cells. Acta Biomater.

[26] Feng Q, Li D, Li Q, Li S, Huang H, Li H (2022). Dynamic nanocomposite microgel assembly with microporosity, injectability, tissue-adhesion, and sustained drug release promotes articular cartilage repair and regeneration. Adv Healthc Mater.

[27] Madry H, Gao L, Rey-Rico A, Venkatesan JK, Muller-Brandt K, Cai X (2020). Thermosensitive hydrogel based on PEO-PPO-PEO poloxamers for a controlled in situ release of recombinant adeno-associated viral vectors for effective gene therapy of cartilage defects. Adv Mater.

[28] Zhang X, Liu S, Wang Z, Luo C, Dai Z, Sun J (2021). Implanted 3D gelatin microcryogel enables low-dose cell therapy for osteoarthritis by preserving the viability and function of umbilical cord MSCs. Chem Eng J.

[29] Yue L, Berman J (2022). What is osteoarthritis?. JAMA.

[30] Katz JN, Arant KR, Loeser RF (2021). Diagnosis and treatment of hip and knee osteoarthritis: a review. JAMA.

[31] Guermazi A, Hayashi D, Roemer FW, Niu J, Quinn EK, Crema MD (2017). Brief report: partial- and full-thickness focal cartilage defects contribute equally to development of new cartilage damage in knee osteoarthritis: the multicenter osteoarthritis study. Arthritis Rheumatol.

[32] Martel-Pelletier J, Barr AJ, Cicuttini FM, Conaghan PG, Cooper C, Goldring MB (2016). Osteoarthritis. Nat Rev Dis Primers.

[33] Mancipe Castro LM, García AJ, Guldberg RE (2021). Biomaterial strategies for improved intra-articular drug delivery. J Biomed Mater Res A.

[34] McCulloch K, Litherland GJ, Rai TS (2017). Cellular senescence in osteoarthritis pathology. Aging Cell.

[35] Shi Y, Hu X, Cheng J, Zhang X, Zhao F, Shi W (2019). A small molecule promotes cartilage extracellular matrix generation and inhibits osteoarthritis development. Nat Commun.

[36] Jeon OH, David N, Campisi J, Elisseeff JH (2018). Senescent cells and osteoarthritis: a painful connection. J Clin Invest.

[37] Li Z, Dai A, Yang M, Chen S, Deng Z, Li L (2022). p38MAPK signaling pathway in osteoarthritis: pathological and therapeutic aspects. J Inflamm Res.

[38] Blanco FJ, Valdes AM, Rego-Pérez I (2018). Mitochondrial DNA variation and the pathogenesis of osteoarthritis phenotypes. Nat Rev Rheumatol.

[39] Choi MC, Jo J, Park J, Kang HK, Park Y (2019). NF-κB signaling pathways in osteoarthritic cartilage destruction. Cells.

[40] Mobasheri A, Rayman MP, Gualillo O, Sellam J, van der Kraan P, Fearon U (2017). The role of metabolism in the pathogenesis of osteoarthritis. Nat Rev Rheumatol.

[41] Hu W, Chen Y, Dou C, Dong S (2021). Microenvironment in subchondral bone: predominant regulator for the treatment of osteoarthritis. Ann Rheum Dis.

[42] Hunter DJ, Bierma-Zeinstra S, Osteoarthritis (2019). Osteoarthritis. Lancet.

[43] Zhao Z, Li Y, Wang M, Zhao S, Zhao Z, Fang J (2020). Mechanotransduction pathways in the regulation of cartilage chondrocyte homoeostasis. J Cell Mol Med.

[44] Sanchez-Lopez E, Coras R, Torres A, Lane NE, Guma M (2022). Synovial inflammation in osteoarthritis progression. Nat Rev Rheumatol.

[45] Tsukasaki M, Takayanagi H (2019). Osteoimmunology: evolving concepts in bone–immune interactions in health and disease. Nat Rev Immunol.

[46] Fan H, Gong JP (2021). Bioinspired underwater adhesives. Adv Mater.

[47] Mehdizadeh M, Yang J (2013). Design strategies and applications of tissue bioadhesives. Macromol Biosci.

[48] Li Y, Cao J, Han S, Liang Y, Zhang T, Zhao H (2018). ECM based injectable thermo-sensitive hydrogel on the recovery of injured cartilage induced by osteoarthritis. Artif Cells Nanomed Biotechnol.

[49] Rey-Rico A, Babicz H, Madry H, Concheiro A, Alvarez Lorenzo C, Cucchiarini M (2017). Supramolecular polypseudorotaxane gels for controlled delivery of rAAV vectors in human mesenchymal stem cells for regenerative medicine. Int J Pharm.

[50] Schalley C. Noncovalent bonding in supramolecular chemistry. Analytical Methods in Supramolecular Chemistry. Weinheim: Wiley-VCH Verlag GmbH & Co. KGaA; 2007. pp. 1–16.

[51] Steiner T (2002). The hydrogen bond in the solid state. Angew Chem Int Ed.

[52] Ge L, Chen S (2020). Recent advances in tissue adhesives for clinical medicine. Polymers.

[53] Li Z, Wang D, Bai H, Zhang S, Ma P, Dong W (2019). Photo-crosslinking strategy constructs adhesive, superabsorbent, and tough PVA‐based hydrogel through controlling the balance of cohesion and adhesion. Macromol Mater Eng.

[54] Li F, Wang A, Wang C (2016). Analysis of friction between articular cartilage and polyvinyl alcohol hydrogel artificial cartilage. J Mater Sci Mater Med.

[55] Branco AC, Oliveira AS, Monteiro I, Nolasco P, Silva DC, Figueiredo-Pina CG (2022). PVA-based hydrogels loaded with diclofenac for cartilage replacement. Gels.

[56] Zhang J, Li B, Zuo J, Gu R, Liu B, Ma C (2021). An engineered protein adhesive with properties of tissue integration and controlled release for efficient cartilage repair. Adv Healthc Mater.

[57] Papadopoulos G, Griffin S, Rathi H, Gupta A, Sharma B, Bavel D (2022). Cost-effectiveness analysis of arthroscopic injection of a bioadhesive hydrogel implant in conjunction with microfracture for the treatment of focal chondral defects of the knee - an australian perspective. J Med Econ.

[58] Lin M, Dai Y, Xia F, Zhang X (2021). Advances in non-covalent crosslinked polymer micelles for biomedical applications. Mater Sci Eng C Mater Biol Appl.

[59] Kim K, Shin M, Koh MY, Ryu JH, Lee MS, Hong S (2015). TAPE: a medical adhesive inspired by a ubiquitous compound in plants. Adv Funct Mater.

[60] Scheiner S (2020). Understanding noncovalent bonds and their controlling forces. J Chem Phys.

[61] Aldred E, Buck C, Vall K. Chapter 3: Bonds found in biological chemistry. In: Pharmacology. Edinburgh: Churchill Livingstone; 2009. pp. 11–9.

[62] Pandey N, Soto-Garcia LF, Liao J, Philippe Z, Nguyen KT, Hong Y (2020). Mussel-inspired bioadhesives in healthcare: design parameters, current trends, and future perspectives. Biomater Sci.

[63] Dhillon S (2011). Fibrin sealant (Evicel® [Quixil®/Crosseal™]). Drugs.

[64] Bouten PJM, Zonjee M, Bender J, Yauw STK, van Goor H, van Hest JCM (2014). The chemistry of tissue adhesive materials. Prog Polym Sci.

[65] Li B, Li F, Ma L, Yang J, Wang C, Wang D (2014). Poly(lactide-co-glycolide)/fibrin gel construct as a 3D model to evaluate gene therapy of cartilage in vivo. Mol Pharm.

[66] Suchaoin W, Bonengel S, Griessinger JA, Pereira de Sousa I, Hussain S, Huck CW (2016). Novel bioadhesive polymers as intra-articular agents: chondroitin sulfate-cysteine conjugates. Eur J Pharm Biopharm.

[67] Mangas-Sanchez J, Sharma M, Cosgrove SC, Ramsden JI, Marshall JR, Thorpe TW (2020). Asymmetric synthesis of primary amines catalyzed by thermotolerant fungal reductive aminases. Chem Sci.

[68] Zou Q, Liu F, Zhao T, Hu X (2021). Reductive amination of ketones/aldehydes with amines using BH3N(C2H5)_3_ as a reductant. Chem Commun (Camb).

[69] Liu X, Yang Y, Niu X, Lin Q, Zhao B, Wang Y (2017). An in situ photocrosslinkable platelet rich plasma-complexed hydrogel glue with growth factor controlled release ability to promote cartilage defect repair. Acta Biomater.

[70] Sigen A, Qian X, Zhou D, Gao Y, Vasquez JM, Greiser U (2017). Hyperbranched PEG-based multi-NHS polymer and bioconjugation with BSA. Polym Chem.

[71] Azim-Zadeh O, Hillebrecht A, Linne U, Marahiel MA, Klebe G, Lingelbach K (2007). Use of biotin derivatives to probe conformational changes in proteins. J Biol Chem.

[72] Li X, Xu Q, Johnson M, Wang X, Lyu J, Li Y (2021). A chondroitin sulfate based injectable hydrogel for delivery of stem cells in cartilage regeneration. Biomater Sci.

[73] Li J, Huang Y, Song J, Li X, Zhang X, Zhou Z (2018). Cartilage regeneration using arthroscopic flushing fluid-derived mesenchymal stem cells encapsulated in a one-step rapid cross-linked hydrogel. Acta Biomater.

[74] Zhang FX, Liu P, Ding W, Meng QB, Su DH, Zhang QC (2021). Injectable mussel-inspired highly adhesive hydrogel with exosomes for endogenous cell recruitment and cartilage defect regeneration. Biomaterials.

[75] Puertas- Bartolomé M, Mora-Boza A, García-Fernández L (2021). Emerging biofabrication techniques: a review on natural polymers for biomedical applications. Polym (Basel).

[76] Badylak SF, Nerem RM (2010). Progress in tissue engineering and regenerative medicine. Proc Natl Acad Sci USA.

[77] Nolan K, Millet Y, Ricordi C, Stabler CL (2008). Tissue engineering and biomaterials in regenerative medicine. Cell Transpl.

[78] Kunjukunju S, Roy A, Ramanathan M, Lee B, Candiello JE, Kumta PN (2013). A layer-by-layer approach to natural polymer-derived bioactive coatings on magnesium alloys. Acta Biomater.

[79] Bhatia S. Natural polymers vs synthetic polymer. In: Bhatia S, editor. Natural polymer drug delivery systems: nanoparticles, plants, and algae. Berlin: Springer; 2016. p. 95–118.

[80] Zhu D, Wang H, Trinh P, Heilshorn SC, Yang F (2017). Elastin-like protein-hyaluronic acid (ELP-HA) hydrogels with decoupled mechanical and biochemical cues for cartilage regeneration. Biomaterials.

[81] Toole BP (2004). Hyaluronan: from extracellular glue to pericellular cue. Nat Rev Cancer.

[82] Burdick JA, Prestwich GD (2011). Hyaluronic acid hydrogels for biomedical applications. Adv Mater.

[83] Hunter DJ (2015). Viscosupplementation for osteoarthritis of the knee. N Engl J Med.

[84] Available from: https://www.aaos.org/globalassets/quality-and-practice-resources/osteoarthritis-of-the-knee/oak3cpg.pdf.

[85] Graça MFP, Miguel SP, Cabral CSD, Correia IJ (2020). Hyaluronic acid-based wound dressings: a review. Carbohydr Polym.

[86] Collins MN, Birkinshaw C (2013). Hyaluronic acid based scaffolds for tissue engineering–a review. Carbohydr Polym.

[87] Tiwari S, Bahadur P (2019). Modified hyaluronic acid based materials for biomedical applications. Int J Biol Macromol.

[88] Chiang CW, Chen CH, Manga YB, Huang SC, Chao KM, Jheng PR (2021). Facilitated and controlled strontium ranelate delivery using GCS-HA nanocarriers embedded into PEGDA coupled with decortication driven spinal regeneration. Int J Nanomedicine.

[89] Wang D, Xu P, Wang S, Li W, Liu W (2020). Rapidly curable hyaluronic acid-catechol hydrogels inspired by scallops as tissue adhesives for hemostasis and wound healing. Eur Polym J.

[90] Chung C, Mesa J, Randolph MA, Yaremchuk M, Burdick JA (2006). Influence of gel properties on neocartilage formation by auricular chondrocytes photoencapsulated in hyaluronic acid networks. J Biomed Mater Res A.

[91] Zhu Y, Wang Y, Sun Y, Shen J, Xu J, Chai Y (2021). In situ self imine-crosslinked nanocomplexes loaded with small noncoding RNA for efficient osteoarthritis attenuation. Chem Eng J.

[92] Loebel C, Szczesny SE, Cosgrove BD, Alini M, Zenobi-Wong M, Mauck RL (2017). Cross-linking chemistry of tyramine-modified hyaluronan hydrogels alters mesenchymal stem cell early attachment and behavior. Biomacromolecules.

[93] Kim BS, Park IK, Hoshiba T, Jiang HL, Choi YJ, Akaike T (2011). Design of artificial extracellular matrices for tissue engineering. Prog Polym Sci.

[94] Levengood SL, Erickson AE, Chang FC, Zhang M (2017). Chitosan-poly(caprolactone) nanofibers for skin repair. J Mater Chem B.

[95] Kong X, Chen L, Li B, Quan C, Wu J (2021). Applications of oxidized alginate in regenerative medicine. J Mater Chem B.

[96] Huebsch N, Arany PR, Mao AS, Shvartsman D, Ali OA, Bencherif SA (2010). Harnessing traction-mediated manipulation of the cell/matrix interface to control stem-cell fate. Nat Mater.

[97] Li W, Wu D, Hu D, Zhu S, Pan C, Jiao Y (2020). Stress-relaxing double-network hydrogel for chondrogenic differentiation of stem cells. Mater Sci Eng C Mater Biol Appl.

[98] Maihöfer J, Madry H, Rey-Rico A, Venkatesan JK, Goebel L, Schmitt G (2021). Hydrogel-guided, rAAV-mediated IGF-I overexpression enables long-term cartilage repair and protection against perifocal osteoarthritis in a large-animal full-thickness chondral defect model at one year in vivo. Adv Mater.

[99] Balakrishnan B, Joshi N, Jayakrishnan A, Banerjee R (2014). Self-crosslinked oxidized alginate/gelatin hydrogel as injectable, adhesive biomimetic scaffolds for cartilage regeneration. Acta Biomater.

[100] Kreller T, Distler T, Heid S, Gerth S, Detsch R, Boccaccini AR (2021). Physico-chemical modification of gelatine for the improvement of 3D printability of oxidized alginate-gelatine hydrogels towards cartilage tissue engineering. Mater Des.

[101] Yan S, Wang T, Feng L, Zhu J, Zhang K, Chen X (2014). Injectable in situ self-cross-linking hydrogels based on poly(L-glutamic acid) and alginate for cartilage tissue engineering. Biomacromolecules.

[102] Islam MM, Shahruzzaman M, Biswas S, Nurus Sakib M, Rashid TU (2020). Chitosan based bioactive materials in tissue engineering applications-a review. Bioact Mater.

[103] Claire Chatelet OD, Alain Domard (2001). Influence of the degree of acetylation on some biological properties of chitosan films. Biomaterials.

[104] Ways M, Lau TM, Khutoryanskiy WM (2018). Chitosan and its derivatives for application in mucoadhesive drug delivery systems. Polym (Basel).

[105] Hoemann CD, Sun J, Légaré A, McKee MD, Buschmann MD (2005). Tissue engineering of cartilage using an injectable and adhesive chitosan-based cell-delivery vehicle. Osteoarthritis Cartilage.

[106] Rahimi M, Mir SM, Baghban R, Charmi G, Plummer CM, Shafiei-Irannejad V (2022). Chitosan-based biomaterials for the treatment of bone disorders. Int J Biol Macromol.

[107] Scognamiglio F, Travan A, Donati I, Borgogna M, Marsich E (2020). A hydrogel system based on a lactose-modified chitosan for viscosupplementation in osteoarthritis. Carbohydr Polym.

[108] Campo GM, Avenoso A, Campo S, D’Ascola A, Ferlazzo AM, Calatroni A (2004). Reduction of DNA fragmentation and hydroxyl radical production by hyaluronic acid and chondroitin-4-sulphate in iron plus ascorbate-induced oxidative stress in fibroblast cultures. Free Radic Res.

[109] Campo GM, Avenoso A, Campo S, D’Ascola A, Traina P, Sama D (2009). Glycosaminoglycans modulate inflammation and apoptosis in LPS-treated chondrocytes. J Cell Biochem.

[110] Henrotin Y, Mathy M, Sanchez C, Lambert C (2010). Chondroitin sulfate in the treatment of osteoarthritis: from in vitro studies to clinical recommendations. Ther Adv Musculoskelet Dis.

[111] Henson FMD, Getgood AMJ, Caborn DM, McIlwraith CW, Rushton N (2012). Effect of a solution of hyaluronic acid-chondroitin sulfate-N-acetyl glucosamine on the repair response of cartilage to single-impact load damage. Am J Vet Res.

[112] Dai C, Zhou Z, Guan Z, Wu Y, Liu Y, He J (2018). A multifunctional metallohydrogel with injectability, self-healing, and multistimulus-responsiveness for bioadhesives. Macromol Mater Eng.

[113] Wang DA, Varghese S, Sharma B, Strehin I, Fermanian S, Gorham J (2007). Multifunctional chondroitin sulphate for cartilage tissue-biomaterial integration. Nat Mater.

[114] García-Coronado JM, Martínez-Olvera L, Elizondo-Omaña RE, Acosta-Olivo CA, Vilchez-Cavazos F, Simental-Mendia LE (2019). Effect of collagen supplementation on osteoarthritis symptoms: a meta-analysis of randomized placebo-controlled trials. Int Orthop.

[115] Koh RH, Jin Y, Kim J, Hwang NS (2020). Inflammation-modulating hydrogels for osteoarthritis cartilage tissue engineering. Cells.

[116] Yang J, Ding C, Tang L, Deng F, Yang Q, Wu H (2020). Novel modification of collagen: realizing desired water solubility and thermostability in a conflict-free way. ACS Omega.

[117] Wang S, Lei J, Yi X, Yuan L, Ge L, Li D (2020). Fabrication of polypyrrole-grafted gelatin-based hydrogel with conductive, self-healing, and injectable properties. ACS Appl Polym Mater.

[118] Satapathy MK, Manga YB, Ostrikov KK, Chiang WH, Pandey A (2020). Microplasma cross-linked graphene oxide-gelatin hydrogel for cartilage reconstructive surgery. ACS Appl Polym Mater Interfaces.

[119] Thangprasert A, Tansakul C, Thuaksubun N, Meesane J (2019). Mimicked hybrid hydrogel based on gelatin/PVA for tissue engineering in subchondral bone interface for osteoarthritis surgery. Mater Des.

[120] Kim J, Lee C, Ryu JH (2021). Adhesive catechol-conjugated hyaluronic acid for biomedical applications: a mini review. Appl Sci.

[121] Kord Forooshani P, Lee BP (2017). Recent approaches in designing bioadhesive materials inspired by mussel adhesive protein. J Polym Sci A Polym Chem.

[122] Hwang DS, Sim SB, Cha HJ (2007). Cell adhesion biomaterial based on mussel adhesive protein fused with RGD peptide. Biomaterials.

[123] Zhang L, Liu M, Zhang Y, Pei R (2020). Recent progress of highly adhesive hydrogels as wound dressings. Biomacromolecules.

[124] Im GI, Kim TK (2020). Regenerative therapy for osteoarthritis: a perspective. Int J Stem Cells.

[125] Ko J, Cha H, Im G (2022). POS0227 mussel adhesive protein-based adhesive to retain stem cells for cartilage regeneration. Ann Rheum Dis.

[126] Spotnitz WD (2010). Fibrin sealant: past, present, and future: a brief review. World J Surg.

[127] Khodakaram-Tafti A, Mehrabani D, Shaterzadeh-Yazdi H (2017). An overview on autologous fibrin glue in bone tissue engineering of maxillofacial surgery. Dent Res J (Isfahan).

[128] Kim YS, Choi YJ, Suh DS, Heo DB, Kim YI, Ryu JS (2015). Mesenchymal stem cell implantation in osteoarthritic knees: is fibrin glue effective as a scaffold?. Am J Sports Med.

[129] Selvakumaran S, Muhamad I, Md Lazim NA. Designing polymeric nanoparticles for targeted drug delivery system. In: Alexander Seifalian AdM, Deepak M, Kalaskar, editors. Nanomedcine. One Central Press; 2014. p. 287–313.

[130] Snetkov P, Zakharova K, Morozkina S, Olekhnovich R, Uspenskaya M (2020). Hyaluronic acid: the influence of molecular weight on structural, physical, physico-chemical, and degradable properties of biopolymer. Polym (Basel).

[131] Reddy MSB, Ponnamma D, Choudhary R, Sadasivuni KK (2021). A comparative review of natural and synthetic biopolymer composite scaffolds. Polym (Basel).

[132] Wu X, He C, Wu Y, Chen X (2016). Synergistic therapeutic effects of Schiff’s base cross-linked injectable hydrogels for local co-delivery of metformin and 5-fluorouracil in a mouse colon carcinoma model. Biomaterials.

[133] Tessmar JK, Göpferich AM (2007). Customized PEG-derived copolymers for tissue-engineering applications. Macromol Biosci.

[134] Wang R, Li J, Chen W, Xu T, Yun S, Xu Z (2017). A biomimetic mussel-inspired ε-Poly-l-lysine hydrogel with robust tissue-anchor and anti-infection capacity. Advd Funct Mater.

[135] Xie T, Ding J, Han X, Jia H, Yang Y, Liang S (2020). Wound dressing change facilitated by spraying zinc ions. Mater Horiz.

[136] Spotnitz WD, Burks S (2012). Hemostats, sealants, and adhesives III: a new update as well as cost and regulatory considerations for components of the surgical toolbox. Transfusion.

[137] Zheng K, Gu Q, Zhou D, Zhou M, Zhang L (2022). Recent progress in surgical adhesives for biomedical applications. Smart Mater Med.

[138] Zhang H, Zhao T, Newland B, Liu W, Wang W, Wang W (2018). Catechol functionalized hyperbranched polymers as biomedical materials. Prog Polym Sci.

[139] Shirwaiker RA, Purser MF, Wysk RA, Narayan R (2014). 6-Scaffolding hydrogels for rapid prototyping based tissue engineering. Rapid prototyping of biomaterials.

[140] Hong J, Oh J, Khan A (2020). Deconstructing poloxamer and poloxamine block copolymers to access poly(ethylene glycol) and poly(propylene oxide)-based thermoresponsive polymers. J Macromol Sci Part A.

[141] Yu J, Qiu H, Yin S, Wang H, Li Y (2021). Polymeric drug delivery system based on pluronics for cancer treatment. Molecules.

[142] Fattahpour S, Shamanian M, Tavakoli N, Fathi M, Sadeghi-Aliabadi H, Sheykhi SR (2020). An injectable carboxymethyl chitosan-methylcellulose-pluronic hydrogel for the encapsulation of meloxicam loaded nanoparticles. Int J Biol Macromol.

[143] Monteiro do Nascimento MH, Ambrosio FN, Ferraraz DC, Windisch-Neto H, Querobino SM, Nascimento-Sales M (2021). Sulforaphane-loaded hyaluronic acid-poloxamer hybrid hydrogel enhances cartilage protection in osteoarthritis models. Mater Sci Eng C Mater Biol Appl.

[144] Lee YH, Chung HJ, Yeo S, Ahn CH, Lee H, Messersmith PB (2010). Thermo-sensitive, injectable, and tissue adhesive sol–gel transition hyaluronic acid/pluronic composite hydrogels prepared from bio-inspired catechol-thiol reaction. Soft Matter.

[145] Bu Y, Ma J, Bei J, Wang S (2019). Surface modification of aliphatic polyester to enhance biocompatibility. Front Bioeng Biotechnol.

[146] Behrens AM, Lee NG, Casey BJ, Srinivasan P, Sikorski MJ, Daristotle JL (2015). Biodegradable-polymer-blend-based surgical sealant with body-temperature-mediated adhesion. Adv Mater.

[147] Tao SC, Huang JY, Gao Y, Li ZX, Wei ZY, Dawes H (2021). Small extracellular vesicles in combination with sleep-related circRNA3503: a targeted therapeutic agent with injectable thermosensitive hydrogel to prevent osteoarthritis. Bioact Mater.

[148] DeMerlis CC, Schoneker DR (2003). Review of the oral toxicity of polyvinyl alcohol (PVA). Food Chem Toxicol.

[149] Gaaz TS, Sulong AB, Akhtar MN, Kadhum AA, Mohamad AB, Al-Amiery AA (2015). Properties and applications of polyvinyl alcohol, halloysite nanotubes and their nanocomposites. Molecules.

[150] Altman R, Bedi A, Manjoo A, Niazi F, Shaw P, Mease P (2019). Anti-inflammatory effects of intra-articular hyaluronic acid: a systematic review. Cartilage.

[151] Altman RD, Manjoo A, Fierlinger A, Niazi F, Nicholls M (2015). The mechanism of action for hyaluronic acid treatment in the osteoarthritic knee: a systematic review. BMC Musculoskelet Disord.

[152] de Lucia O, Murgo A, Pregnolato F, Pontikaki I, De Souza M, Sinelli A (2020). Hyaluronic acid injections in the treatment of osteoarthritis secondary to primary inflammatory rheumatic diseases: a systematic review and qualitative synthesis. Adv Ther.

[153] Gambaro FM, Ummarino A, Torres Andón F, Ronzoni F, Di Matteo B, Kon E (2021). Drug delivery systems for the treatment of knee osteoarthritis: a systematic review of in vivo studies. Int J Mol Sci.

[154] Teng B, Zhang S, Pan J, Zeng Z, Chen Y, Hei Y (2021). A chondrogenesis induction system based on a functionalized hyaluronic acid hydrogel sequentially promoting hMSC proliferation, condensation, differentiation, and matrix deposition. Acta Biomater.

[155] Shi W, Fang F, Kong Y, Greer SE, Kuss M, Liu B, et al. Dynamic hyaluronic acid hydrogel with covalent linked gelatin as an anti-oxidative bioink for cartilage tissue engineering. Biofabrication. 2021. 10.1088/1758-5090/ac42de. 10.1088/1758-5090/ac42de 34905737

[156] Tanaka T, Matsushita T, Nishida K, Takayama K, Nagai K, Araki D (2019). Attenuation of osteoarthritis progression in mice following intra-articular administration of simvastatin-conjugated gelatin hydrogel. J Tissue Eng Regen Med.

[157] Santos VHD, Pfeifer JPH, Souza JB, Stievani FC, Hussni CA, Golim MA (2019). Evaluation of alginate hydrogel encapsulated mesenchymal stem cell migration in horses. Res Vet Sci.

[158] Reginster JY, Veronese N (2021). Highly purified chondroitin sulfate: a literature review on clinical efficacy and pharmacoeconomic aspects in osteoarthritis treatment. Aging Clin Exp Res.

[159] Mishra S, Ganguli M (2021). Functions of, and replenishment strategies for, chondroitin sulfate in the human body. Drug Discov Today.

[160] Xu J, Feng Q, Lin S, Yuan W, Li R, Li J (2019). Injectable stem cell-laden supramolecular hydrogels enhance in situ osteochondral regeneration via the sustained co-delivery of hydrophilic and hydrophobic chondrogenic molecules. Biomaterials.

[161] Martel-Pelletier J, Kwan Tat S, Pelletier JP (2010). Effects of chondroitin sulfate in the pathophysiology of the osteoarthritic joint: a narrative review. Osteoarthritis Cartilage.

[162] Radhakrishnan J, Manigandan A, Chinnaswamy P, Subramanian A, Sethuraman S (2018). Gradient nano-engineered in situ forming composite hydrogel for osteochondral regeneration. Biomaterials.

[163] Richardson BM, Wilcox DG, Randolph MA, Anseth KS (2019). Hydrazone covalent adaptable networks modulate extracellular matrix deposition for cartilage tissue engineering. Acta Biomater.

[164] Ding M, Christian Danielsen C, Hvid I (2005). Effects of hyaluronan on three-dimensional microarchitecture of subchondral bone tissues in guinea pig primary osteoarthrosis. Bone.

[165] Zhou D, Li S, Pei M, Yang H, Gu S, Tao Y (2020). Dopamine-modified hyaluronic acid hydrogel adhesives with fast-forming and high tissue adhesion. ACS Appl Mater Interfaces.

[166] Behrendt P, Ladner Y, Stoddart MJ, Lippross S, Alini M, Eglin D (2020). Articular joint-simulating mechanical load activates endogenous TGF-β in a highly cellularized bioadhesive hydrogel for cartilage repair. Am J Sports Med.

[167] Aldana AA, Abraham GA (2017). Current advances in electrospun gelatin-based scaffolds for tissue engineering applications. Int J Pharm.

[168] Salamon A, van Vlierberghe S, van Nieuwenhove I, Baudisch F, Graulus GJ, Benecke V (2014). Gelatin-based hydrogels promote chondrogenic differentiation of human adipose tissue-derived mesenchymal stem cells in vitro. Mater (Basel).

[169] Goldring MB (2012). Chondrogenesis, chondrocyte differentiation, and articular cartilage metabolism in health and osteoarthritis. Ther Adv Musculoskelet Dis.

[170] Sulaiman S, Chowdhury SR, Fauzi MB, Rani RA, Yahaya NHM, Tabata Y (2020). 3D culture of MSCs on a gelatin microsphere in a dynamic culture system enhances chondrogenesis. Int J Mol Sci.

[171] Igarashi T, Iwasaki N, Kawamura D, Tsukuda Y, Kasahara Y, Todoh M (2012). Therapeutic effects of intra-articular ultrapurified low endotoxin alginate administration on experimental osteoarthritis in rabbits. Cartilage.

[172] Ghanbari M, Salavati-Niasari M, Mohandes F, Firouzi Z (2022). Modified silicon carbide NPs reinforced nanocomposite hydrogels based on alginate-gelatin by with high mechanical properties for tissue engineering. Arab J Chem.

[173] Wong CC, Lu CX, Cho EC, Lee PW, Chi NW, Lin PY (2022). Calcium peroxide aids tyramine-alginate gel to crosslink with tyrosinase for efficient cartilage repair. Int J Biol Macromol.

[174] Levillain A, Magoariec H, Boulocher C, Decambron A, Viateau V, Hoc T (2017). Effects of a viscosupplementation therapy on rabbit menisci in an anterior cruciate ligament transection model of osteoarthritis. J Biomech.

[175] Beadle C, Howie C, Nuki G (2010). Oarsi recommendations for the management of hip and knee osteoarthritis: which treatments are being used? Audit of patients coming to arthroplasty in Scotland. Osteoarthritis Cartilage.

[176] Zheng L, Zhang Z, Sheng P, Mobasheri A (2021). The role of metabolism in chondrocyte dysfunction and the progression of osteoarthritis. Ageing Res Rev.

[177] Veronese N, Cooper C, Reginster JY, Hochberg M, Branco J, Bruyère O (2019). Type 2 diabetes mellitus and osteoarthritis. Semin Arthritis Rheum.

[178] Little CJ, Kulyk WM, Chen X (2014). The effect of chondroitin sulphate and hyaluronic acid on chondrocytes cultured within a fibrin-alginate hydrogel. J Funct Biomater.

[179] Mohamed AL, Elmotasem H, Salama AAA (2020). Colchicine mesoporous silica nanoparticles/hydrogel composite loaded cotton patches as a new encapsulator system for transdermal osteoarthritis management. Int J Biol Macromol.

[180] Tamura T, Higuchi Y, Kitamura H, Murao N, Saitoh R, Morikawa T (2016). Novel hyaluronic acid-methotrexate conjugate suppresses joint inflammation in the rat knee: efficacy and safety evaluation in two rat arthritis models. Arthritis Res Ther.

[181] Zhang Z, Wei X, Gao J, Zhao Y, Zhao Y, Guo L (2016). Intra-articular injection of cross-linked hyaluronic acid-dexamethasone hydrogel attenuates osteoarthritis: an experimental study in a rat model of osteoarthritis. Int J Mol Sci.

[182] Khan D, Qindeel M, Ahmed N, Asad MI, Shah KU, Asim Ur R (2021). Development of an intelligent, stimuli-responsive transdermal system for efficient delivery of Ibuprofen against rheumatoid arthritis. Int J Pharm.

[183] Park JW, Yun YP, Park K, Lee JY, Kim HJ, Kim SE (2016). Ibuprofen-loaded porous microspheres suppressed the progression of monosodium iodoacetate-induced osteoarthritis in a rat model. Colloids Surf B Biointerfaces.

[184] Chu M, Wu P, Hong M, Zeng H, Wong CK, Feng Y (2021). Lingzhi and San-Miao-San with hyaluronic acid gel mitigate cartilage degeneration in anterior cruciate ligament transection induced osteoarthritis. J Orthop Translat.

[185] Li X, Ding J, Zhang Z, Yang M, Yu J, Wang J (2016). Kartogenin-incorporated thermogel supports stem cells for significant cartilage regeneration. ACS Appl Mater Interfaces.

[186] Liu C, Li T, Yang Z, Liu D, Li Y, Zhou Z (2018). Kartogenin enhanced chondrogenesis in cocultures of chondrocytes and bone mesenchymal stem cells. Tissue Eng Part A.

[187] Chen H, Qin Z, Zhao J, He Y, Ren E, Zhu Y (2019). Cartilage-targeting and dual MMP-13/pH responsive theranostic nanoprobes for osteoarthritis imaging and precision therapy. Biomaterials.

[188] Abou-ElNour M, Soliman ME, Skouras A, Casettari L, Geneidi AS, Ishak RAH (2020). Microparticles-in-thermoresponsive/bioadhesive hydrogels as a novel integrated platform for effective intra-articular delivery of triamcinolone acetonide. Mol Pharm.

[189] McAlindon TE, LaValley MP, Harvey WF, Price LL, Driban JB, Zhang M (2017). Effect of intra-articular triamcinolone vs saline on knee cartilage volume and pain in patients with knee osteoarthritis: a randomized clinical trial. JAMA.

[190] Oh GW, Kim SC, Kim TH, Jung WK (2021). Characterization of an oxidized alginate-gelatin hydrogel incorporating a COS-salicylic acid conjugate for wound healing. Carbohydr Polym.

[191] Tsubosaka M, Kihara S, Hayashi S, Nagata J, Kuwahara T, Fujita M (2020). Gelatin hydrogels with eicosapentaenoic acid can prevent osteoarthritis progression in vivo in a mouse model. J Orthop Res.

[192] Jeong SH, Kim M, Kim TY, Kim H, Ju JH, Hahn SK (2020). Supramolecular injectable hyaluronate hydrogels for cartilage tissue regeneration. ACS Appl Bio Mater.

[193] Li H, Jin Y, Zhao Y, Li W, He Z, Zhang Q (2021). Targeted cell therapy for partial-thickness cartilage defects using membrane modified mesenchymal stem cells by transglutaminase 2. Biomaterials.

[194] Lee WS, Kim HJ, Kim KI, Kim GB, Jin W (2019). Intra-articular injection of autologous adipose tissue-derived mesenchymal stem cells for the treatment of knee osteoarthritis: a phase IIb, randomized, placebo-controlled clinical trial. Stem Cells Transl Med.

[195] Wang CZ, Eswaramoorthy R, Lin TH, Chen CH, Fu YC, Wang CK (2018). Enhancement of chondrogenesis of adipose-derived stem cells in HA-PNIPAAm-CL hydrogel for cartilage regeneration in rabbits. Sci Rep.

[196] Park YB, Ha CW, Lee CH, Yoon YC, Park YG (2017). Cartilage regeneration in osteoarthritic patients by a composite of allogeneic umbilical cord blood-derived mesenchymal stem cells and hyaluronate hydrogel: results from a clinical trial for safety and proof-of-concept with 7 years of extended follow-up. Stem Cells Transl Med.

[197] Wu KC, Chang YH, Liu HW, Ding DC (2019). Transplanting human umbilical cord mesenchymal stem cells and hyaluronate hydrogel repairs cartilage of osteoarthritis in the minipig model. Ci Ji Yi Xue Za Zhi.

[198] Man Z, Hu X, Liu Z, Huang H, Meng Q, Zhang X (2016). Transplantation of allogenic chondrocytes with chitosan hydrogel-demineralized bone matrix hybrid scaffold to repair rabbit cartilage injury. Biomaterials.

[199] Li X, Li S, Qian J, Chen Y, Zhou Y, Fu P (2021). Early efficacy of type i collagen-based matrix-assisted autologous chondrocyte transplantation for the treatment of articular cartilage lesions. Front Bioeng Biotechnol.

[200] Zhou T, Li X, Li G, Tian T, Lin S, Shi S (2017). Injectable and thermosensitive TGF-β1-loaded PCEC hydrogel system for in vivo cartilage repair. Sci Rep.

[201] Han F, Zhou F, Yang X, Zhao J, Zhao Y, Yuan X (2015). A pilot study of conically graded chitosan-gelatin hydrogel/PLGA scaffold with dual-delivery of TGF-β1 and BMP-2 for regeneration of cartilage-bone interface. J Biomed Mater Res B Appl Biomater.

[202] Tarafder S, Gulko J, Sim KH, Yang J, Cook JL, Lee CH (2018). Engineered healing of avascular meniscus tears by stem cell recruitment. Sci Rep.

[203] Akkiraju H, Bonor J, Nohe A (2017). CK2.1, a novel peptide, induces articular cartilage formation in vivo. J Orthop Res.

[204] Kung FC (2018). Injectable collagen/RGD systems for bone tissue engineering applications. Biomed Mater Eng.

[205] Moreira Teixeira LS, Leijten JCH, Wennink JWH, Chatterjea AG, Feijen J, van Blitterswijk CA (2012). The effect of platelet lysate supplementation of a dextran-based hydrogel on cartilage formation. Biomaterials.

[206] Raeissadat SA, Ghazi Hosseini P, Bahrami MH, Salman Roghani R, Fathi M, Gharooee Ahangar A (2021). The comparison effects of intra-articular injection of platelet Rich plasma (PRP), plasma Rich in Growth factor (PRGF), Hyaluronic Acid (HA), and ozone in knee osteoarthritis; a one year randomized clinical trial. BMC Musculoskelet Disord.

[207] Zhao R, Peng X, Li Q, Song W (2014). Effects of phosphorylatable short peptide-conjugated chitosan-mediated IL-1Ra and igf-1 gene transfer on articular cartilage defects in rabbits. PLoS ONE.

[208] Rey-Rico A, Venkatesan JK, Frisch J, Rial-Hermida I, Schmitt G, Concheiro A (2015). PEO-PPO-PEO micelles as effective rAAV-mediated gene delivery systems to target human mesenchymal stem cells without altering their differentiation potency. Acta Biomater.

[209] Haseeb A, Kc R, Angelozzi M, de Charleroy C, Rux D, Tower RJ (2021). SOX9 keeps growth plates and articular cartilage healthy by inhibiting chondrocyte dedifferentiation/osteoblastic redifferentiation. Proc Natl Acad Sci U S A.

[210] Ledo AM, Senra A, Rilo-Alvarez H, Borrajo E, Vidal A, Alonso MJ (2020). mRNA-activated matrices encoding transcription factors as primers of cell differentiation in tissue engineering. Biomaterials.

[211] Liu X, Yang Y, Li Y, Niu X, Zhao B, Wang Y (2017). Integration of stem cell-derived exosomes with in situ hydrogel glue as a promising tissue patch for articular cartilage regeneration. Nanoscale.

[212] Zhang S, Chuah SJ, Lai RC, Hui JHP, Lim SK, Toh WS (2018). MSC exosomes mediate cartilage repair by enhancing proliferation, attenuating apoptosis and modulating immune reactivity. Biomaterials.

[213] Bhala N, Emberson J, Merhi A, Abramson S, Arber N, Baron JA (2013). Vascular and upper gastrointestinal effects of non-steroidal anti-inflammatory drugs: meta-analyses of individual participant data from randomised trials. Lancet.

[214] Magni A, Agostoni P, Bonezzi C, Massazza G, Mene P, Savarino V (2021). Management of osteoarthritis: expert opinion on NSAIDs. Pain Ther.

[215] Sánchez A, Schimmang T, García-Sancho J (2012). Cell and tissue therapy in regenerative medicine. Adv Exp Med Biol.

[216] Sulaiman SB, Idrus RBH, Hwei NM (2020). Gelatin microsphere for cartilage tissue engineering: current and future strategies. Polym (Basel).

[217] Kudva AK, Dikina AD, Luyten FP, Alsberg E, Patterson J (2019). Gelatin microspheres releasing transforming growth factor drive in vitro chondrogenesis of human periosteum derived cells in micromass culture. Acta Biomater.

[218] Inoue A, Takahashi KA, Arai Y, Tonomura H, Sakao K, Saito M (2006). The therapeutic effects of basic fibroblast growth factor contained in gelatin hydrogel microspheres on experimental osteoarthritis in the rabbit knee. Arthritis Rheum.

[219] Park E, Hart ML, Rolauffs B, Stegemann JP, Annamalai T (2020). Bioresponsive microspheres for on-demand delivery of anti-inflammatory cytokines for articular cartilage repair. J Biomed Mater Res A.

[220] He Y, Mu C, Shen X, Yuan Z, Liu J, Chen W (2018). Peptide LL-37 coating on micro-structured titanium implants to facilitate bone formation in vivo via mesenchymal stem cell recruitment. Acta Biomater.

[221] Liu P, Li M, Yu H, Fang H, Yin J, Zhu D (2021). Biphasic CK2.1-coated β-glycerophosphate chitosan/LL37-modified layered double hydroxide chitosan composite scaffolds enhance coordinated hyaline cartilage and subchondral bone regeneration. Chem Eng J.

[222] Fang J, Wang X, Jiang W, Zhu Y, Hu Y, Zhao Y (2020). Platelet-rich plasma therapy in the treatment of diseases associated with orthopedic injuries. Tissue Eng Part B Rev.

[223] Andia I, Maffulli N (2013). Platelet-rich plasma for managing pain and inflammation in osteoarthritis. Nat Rev Rheumatol.

[224] Grol MW, Lee BH (2018). Gene therapy for repair and regeneration of bone and cartilage. Curr Opin Pharmacol.

[225] Raisin S, Belamie E, Morille M (2016). Non-viral gene activated matrices for mesenchymal stem cells based tissue engineering of bone and cartilage. Biomaterials.

[226] Yang B, Chen Y, Shi J (2019). Exosome biochemistry and advanced nanotechnology for next-generation theranostic platforms. Adv Mater.

[227] He C, Zheng S, Luo Y, Wang B (2018). Exosome theranostics: biology and translational medicine. Theranostics.

[228] Thakur A, Parra DC, Motallebnejad P, Brocchi M, Chen HJ (2022). Exosomes: small vesicles with big roles in cancer, vaccine development, and therapeutics. Bioact Mater.

[229] Wu J, Kuang L, Chen C, Yang J, Zeng WN, Li T (2019). Mir-100-5p-abundant exosomes derived from infrapatellar fat pad MSCs protect articular cartilage and ameliorate gait abnormalities via inhibition of mTOR in osteoarthritis. Biomaterials.

[230] Ayala-Mar S, Donoso-Quezada J, Gallo-Villanueva RC, Perez-Gonzalez VH, Gonzalez-Valdez J (2019). Recent advances and challenges in the recovery and purification of cellular exosomes. Electrophoresis.

[231] Guérin G, Pujol N (2020). Repair of large condylar osteochondral defects of the knee by collagen scaffold. Minimum two-year outcomes. Orthop Traumatol Surg Res.

[232] Wang D, Nawabi DH, Krych AJ, Jones KJ, Nguyen J, Elbuluk AM (2021). Synthetic biphasic scaffolds versus microfracture for articular cartilage defects of the knee: a retrospective comparative study. Cartilage.

[233] Wong SHM, Lim SS, Tiong TJ, Show PL, Zaid HFM, Loh HS (2020). Preliminary in vitro evaluation of chitosan-graphene oxide scaffolds on osteoblastic adhesion, proliferation, and early differentiation. Int J Mol Sci.

[234] Petri M, Broese M, Simon A, Liodakis E, Ettinger M, Guenther D (2013). CaReS (MACT) versus microfracture in treating symptomatic patellofemoral cartilage defects: a retrospective matched-pair analysis. J Orthop Sci.

[235] Kreuz PC, Kalkreuth RH, Niemeyer P, Uhl M, Erggelet C (2019). Long-term clinical and MRI results of matrix-assisted autologous chondrocyte implantation for articular cartilage defects of the knee. Cartilage.

[236] Brix MO, Stelzeneder D, Chiari C, Koller U, Nehrer S, Dorotka R (2014). Treatment of full-thickness chondral defects with hyalograft c in the knee: long-term results. Am J Sports Med.

[237] Mehta S, He T, Bajpayee AG (2021). Recent advances in targeted drug delivery for treatment of osteoarthritis. Curr Opin Rheumatol.

[238] Evans CH, Kraus VB, Setton LA (2014). Progress in intra-articular therapy. Nat Rev Rheumatol.

[239] Identifier. NCT03005873 [Available from: https://clinicaltrials.gov/ct2/show/NCT03005873?cond=NCT03005873&draw=2&rank=1].

[240] Identifier. NCT03754049 [Available from: https://clinicaltrials.gov/ct2/show/NCT03754049?cond=NCT03754049&draw=2&rank=1].

[241] Identifier. NCT04123561 [Available from: https://clinicaltrials.gov/ct2/show/NCT04123561?cond=NCT04123561&draw=2&rank=1].

[242] Malone A, Price J, Price N, Peck V, Getgood A, Petrella R (2021). Safety and pharmacokinetics of EP-104IAR (sustained-release fluticasone propionate) in knee osteoarthritis: a randomized, double-blind, placebo-controlled phase 1 trial. Osteoarthr Cartil Open.

[243] Identifier. NCT03209362 [Available from: https://clinicaltrials.gov/ct2/show/NCT03209362?cond=NCT03209362&draw=2&rank=1].

[244] Identifier. NCT02417610 [Available from: https://clinicaltrials.gov/ct2/show/NCT02417610?cond=NCT02417610&draw=2&rank=1].

[245] Identifier. NCT04589611 [Available from: https://clinicaltrials.gov/ct2/show/NCT04589611?cond=NCT04589611&draw=1&rank=1].

[246] Yang SY, O’Cearbhaill ED, Sisk GC, Park KM, Cho WK, Villiger M (2013). A bio-inspired swellable microneedle adhesive for mechanical interlocking with tissue. Nat Commun.

[247] Ma Y, Ma S, Wu Y, Pei X, Gorb SN, Wang Z (2018). Remote control over underwater dynamic attachment/detachment and locomotion. Adv Mater.

[248] Li J, Celiz AD, Yang J, Yang Q, Wamala I, Whyte W (2017). Tough adhesives for diverse wet surfaces. Science.

[249] Yuk H, Varela CE, Nabzdyk CS, Mao X, Padera RF, Roche ET (2019). Dry double-sided tape for adhesion of wet tissues and devices. Nature.

[250] Nakayama A, Kakugo A, Gong JP, Osada Y, Takai M, Erata T (2004). High mechanical strength double-network hydrogel with bacterial cellulose. Adv Funct Mater.

[251] Nonoyama TGJP (2015). Double-network hydrogel and its potential biomedical application: a review. Proc Inst Mech Eng H.

[252] Cai L, Dewi RE, Heilshorn SC (2015). Injectable hydrogels with in situ double network formation enhance retention of transplanted stem cells. Adv Funct Mater.

[253] Ding X, Wang Y, Liu J, Zhang P, Li G, Sun T (2021). Injectable in situ forming double-network hydrogel to enhance transplanted cell viability and retention. Chem Mater.

[254] Zhang C, Huang J, Zhang J, Liu S, Cui M, An B (2019). Engineered bacillus subtilis biofilms as living glues. Mater Today.

[255] Klimak M, Nims RJ, Pferdehirt L, Collins KH, Harasymowicz NS, Oswald SJ (2021). Immunoengineering the next generation of arthritis therapies. Acta Biomater.

[256] Zhu M, Wei K, Lin S, Chen X, Wu CC, Li G (2018). Bioadhesive polymersome for localized and sustained drug delivery at pathological sites with harsh enzymatic and fluidic environment via supramolecular host-guest complexation. Small.

[257] Wang W, Li B, Li Y, Jiang Y, Ouyang H, Gao C (2010). In vivo restoration of full-thickness cartilage defects by poly(lactide-co-glycolide) sponges filled with fibrin gel, bone marrow mesenchymal stem cells and DNA complexes. Biomaterials.

[258] Guo J, Sun W, Kim JP, Lu X, Li Q, Lin M (2018). Development of tannin-inspired antimicrobial bioadhesives. Acta Biomater.

[259] Lu X, Shi S, Li H, Gerhard E, Lu Z, Tan X (2020). Magnesium oxide-crosslinked low-swelling citrate-based mussel-inspired tissue adhesives. Biomaterials.

[260] Guo J, Kim GB, Shan D, Kim JP, Hu J, Wang W (2017). Click chemistry improved wet adhesion strength of mussel-inspired citrate-based antimicrobial bioadhesives. Biomaterials.

[261] Mohd Yunus MH, Lee Y, Nordin A, Chua KH (2022). Bt Hj Idrus R. Remodeling osteoarthritic articular cartilage under hypoxic conditions. Int J Mol Sci.

[262] Berenbaum F, Griffin TM, Liu-Bryan R (2017). Review: metabolic regulation of inflammation in osteoarthritis. Arthritis Rheumatol.

[263] Zhang C, Lin Y, Yan CH, Zhang W (2022). Adipokine signaling pathways in osteoarthritis. Front Bioeng Biotechnol.

[264] Reseland JE, Syversen U, Bakke I (2001). Leptin is expressed in and secreted from primary cultures of human osteoblasts. J Bone Miner Res.

